# Myostatin Exhibits an Evolutionarily Conserved Circadian Pattern in Skeletal Muscles

**DOI:** 10.1002/jcsm.70130

**Published:** 2025-11-24

**Authors:** Xiangpeng Liu, Changyou Song, Yan Xiong, Jinxin Gu, Lianxin Wu, Taole Liu, Xiyue Chen, Hui Shu, Yingying Dong, Tizhong Shan, Sheng Wang, Yucheng Zhu, Tongxing Song, Lei Fu, Yaqiu Lin, Can Liu, Ruiqi Zheng, Xiao Zhao, Hongxia Li, Yong Xu, Shihuan Kuang, Han Wang, Bin Guo, Pao Xu, Zhihao Jia

**Affiliations:** ^1^ Cambridge‐Suda Genomic Resource Center Suzhou Medical College, Soochow University Suzhou China; ^2^ Institues of Biomedical Sciences Inner Mongolia University Hohhot China; ^3^ Freshwater Fisheries Research Center Chinese Academy of Fishery Sciences Wuxi China; ^4^ Key Laboratory of Qinghai‐Tibetan Plateau Animal Genetic Resource Reservation and Utilization, Ministry of Education Southwest Minzu University Chengdu China; ^5^ Wisdom Lake Academy of Pharmacy Xi'an Jiaotong‐Liverpool University Suzhou China; ^6^ Center for Circadian Clocks, Soochow University, and School of Basic Medical Sciences, Suzhou Medical College Suzhou Jiangsu China; ^7^ Department of Animal Sciences Purdue University West Lafayette Indiana USA; ^8^ College of Animal Sciences Zhejiang University Hangzhou China; ^9^ State Key Laboratory of Biocontrol, School of Life Sciences Sun Yat‐Sen University Guangzhou China; ^10^ College of Animal Science and Technology Huazhong Agricultural University Wuhan China; ^11^ Departmen of Orthopedics, the Third Affiliated Hospital of Southern Medical University Guangzhou China; ^12^ Med‐X Institute, Center for Immunological and Metabolic Diseases and Department of Endocrinology, First Affiliated Hospital of Xi'an Jiaotong University Xi'an Jiaotong University Xi'an China; ^13^ Orthopaedic Institute Suzhou Medical College, Soochow University Suzhou China; ^14^ Department of Orthopedic Surgery and Department of Cell Biology Duke University School of Medicine Durham North Carolina USA

**Keywords:** circadian clock, myostatin, skeletal muscle

## Abstract

**Introduction:**

Myostatin (MSTN), a transforming growth factor‐beta (TGF‐β) superfamily member, is an evolutionarily conserved negative regulator of skeletal muscle mass. Loss of MSTN commonly promotes augmentation in skeletal muscle mass in all animal species examined. Recent studies have demonstrated that circadian clock proteins play a critical role in the regulation of muscle mass and function, in part by modulating the expression of key muscle‐related genes. While myostatin has an important role in sustaining skeletal muscle protein turnover, it is unknown if circadian clock proteins regulate myostatin in a circadian pattern.

**Methods:**

We analysed time‐course muscle samples from 16 animal species ranging from *Caenorhabditis elegans* to humans and examined the rhythmic expression pattern of *Mstn*. We also used various circadian clock deficient models such as muscle‐specific *Bmal1* knockout, *Per1/Per2* double knockout, genetic knockout of *per0* and *tim0* genes in fruit flies, *clocka* gene in zebrafish and environmental perturbation.

**Results:**

Both mRNA and protein of MSTN exhibit rhythmic expression patterns in a variety of animal species ranging from *Caenorhabditis elegans* (*C. elegans*) to humans. The rhythmicity of *Mstn* orthologs in muscle is evolutionarily conserved along with their sequence evolution in *C. elegans* , *Drosophila melanogaster* , *Crustacea*, fish and mammals including mice (mRNA: amplitude = 0.188, *p* < 0.0001; protein: amplitude = 0.255, *p* < 0.05), goats, pigs and humans. In murine skeletal muscle, rhythmic expression of *Mstn* is synchronized with the core circadian genes, *Per2*. We then constructed a muscle‐specific *Bmal1* knockout mouse model (*Bmal1* ^*MKO*^). Notably, *Bmal1* ^*MKO*^ mice had increased body weight (29.30 ± 0.85 vs. 32.16 ± 0.79, *p* < 0.05) and lean mass (WT 23.33 ± 0.35 vs. 25.35 ± 0.45, *p* < 0.01), while the difference in lean mass at 12 weeks of age (~1.996 g) closely matches the difference in total body weight (~ 2.000 g). Muscle‐specific *Bmal1* knockout reduced the mRNA and protein levels of *mstn*/MSTN by ~ 50%. In addition, disruption of the circadian clock by constant light or *Per1/Per2* double knockout also abolishes the rhythmicity of *Mstn*. Similarly, genetic knockout of *per0* and *tim0* genes in fruit flies, *clocka* gene in zebrafish (*mstna*: *p* < 0.01 vs. *p* = 0.6397) and environmental perturbation ( *Aplodinotus grunniens* , *mstn1*: *p* < 0.0001 vs. *p* = 0.04; *mstn2*: *p* < 0.05 vs. *p* = 0.06) all alter *Mstn* oscillation profoundly.

**Conclusions:**

These findings reveal an evolutionarily conserved rhythmic expression pattern of *Mstn* in skeletal muscles.

## 1 Introduction

Circadian rhythm is an inherent and evolutionarily conserved feature across cell function and animal behaviour [1, 2]. The circadian timing system, through transcription‐translation negative feedback loops, facilitates rhythmic transcription of core clock factors [3]. In simple terms, the heterodimer comprising brain and muscle ARNT‐like 1 (BMAL1) and circadian locomotor output cycles kaput (CLOCK) functions as a transcriptional stimulator. This heterodimer governs the transcription of key circadian clock genes such as *Period* (*Per*) and *Cryptochrome* (*Cry*), which encode the repressors of the BMAL1:CLOCK heterodimer activity, resulting in approximately 24‐h rhythmic oscillations [4]. Circadian rhythm exerts significant effects over the primary physiological performances and biological properties of skeletal muscle, including sarcomere operation, metabolic flexibility and protein turnover [5]. A recent study with chromatin immunoprecipitation with sequencing (ChIP‐seq) revealed that the BMAL1:CLOCK heterodimer binds to approximately 5000 genomic sites in skeletal muscle, potentially regulating the expression of these actively transcribed genes involved in muscle mass and function [6, 7, 8, 9].

Skeletal muscle constitutes the largest tissue mass and the primary protein reservoir in the body, requiring precise regulation of protein synthesis and degradation to maintain protein turnover [10, 11]. It is tightly regulated, akin to the circadian clock, ensuring the maintenance of muscle mass and function or enabling response to environmental stimuli [12, 13]. The synthesis rate of protein largely depends on the quantity and quality of ribosomes [14]. Numerous studies reinforce the association between the circadian clock and the regulation of protein synthesis. For instance, the circadian clock has been shown to govern the transcription of ribosomal protein mRNAs and ribosomal RNAs directly in the liver [15, 16] and rhythmic expression of genes involved in mTOR (the mechanistic target of rapamycin) signaling, a pivotal determinant of translational efficiency [17]. On the other hand, the degradation of protein in skeletal muscle is predominantly executed by the ubiquitin‐proteasome system, the autophagy‐lysosome pathway and endopeptidases [18, 19]. In concert, these systems function to eliminate damaged or long‐lasting proteins in the skeletal muscle, thus preserving optimal cellular physiology. Several components of the atrophic pathway, such as muscle RING finger 1 (MuRF1) and Atrogin‐1, are reportedly under circadian regulation and can be directly upregulated by the BMAL1‐CLOCK heterodimer [20].

Myostatin, belonging to the transforming growth factor‐beta (TGF‐β) family, is the most well‐known negative modulator of skeletal muscle mass [21], due to the significant hypermuscular myostatin‐deficient cattle [22]. Myostatin on the one hand promotes protein degradation in skeletal muscle by enhancing the expression of *MuRF1* and *Atrogin‐1* [23, 24] and on the other hand inhibits protein synthesis by inhibiting the Akt/mTOR pathway [25]. Despite Myostatin's pivotal role in sustaining skeletal muscle protein turnover and the known interplay between the circadian clock and protein homeostasis, whether the expression of myostatin exhibits circadian variation is unknown.

## 2 Methods

### 2.1 Mouse Strains and Housing

All mice used in this study were male C57BL/6 mice aged 12 weeks and were bred and housed in the specific pathogen‐free (SPF) grade animal facility of CAM‐SU (Suzhou, China). The animals had ad libitum (in an 8:00 to 20:00 light and dark cycle) access to acidified water and standard rodent chow, which was irradiated and autoclaved. All procedures involving animal maintenance and experimentation were conducted in accordance with the animal protocols (ZJ‐2021‐1), which were approved by the Institutional Animal Care and Use Committee of CAM‐SU on December 24, 2021.

To generate mature skeletal muscle‐specific *Bmal1* knockout (*Bmal1* ^*MKO*^) mice, *Bmal1* ^*fl/fl*^ animals were crossed with transgenic mice expressing Cre recombinase under the control of the mature skeletal muscle and cardiac muscle promoter (*Ckmm‐Cre*), and the resulting offspring were maintained in a heterozygous breeding scheme. Cre‐negative littermates were used as controls for all experiments. *Per1/Per2* double knockout (*Per1/Per2* DKO) mice were generated by crossing *Per1* and *Per2* global knockout mouse models.

### 2.2 Sample Collection

All animal studies used standardized time definitions: zeitgeber time (ZT) refers to time under a light–dark cycle (with ZT0 defined as lights on), while circadian time (CT) is defined by the organism's internal clock and was used to describe time points in constant conditions (e.g., constant darkness or light) in this study.

Mice within the control group were housed with a controlled light cycle of 12‐h light and 12‐h dark (lights on at ZT0, corresponding to 8:00 local time; off at ZT12, corresponding to 20:00 local time). Starting from ZT0 (8:00 local time), skeletal muscle tissue samples were collected from genetically modified mice and respective control littermates every 4 h: ZT0, ZT4, ZT8, ZT12, ZT16 and ZT20 corresponding to 8:00–4:00 (the next day) local time. Samples collected from mice under constant dark (dark/dark, DD) or constant light (light/light, LL) were described as follows: CT0, CT4, CT8, CT12, CT16 and CT20, corresponding to 8:00–4:00 (the next day) local time. All samples were immediately frozen in liquid nitrogen and stored at −80°C until further analysis. This sampling procedure was performed in at least three independent experiments with biological replicates to ensure the reproducibility and reliability of the data for subsequent biochemical analyses.

Human deltoid muscle samples were collected by authors from the Third Affiliated Hospital of Southern Medical University under a protocol number 2023‐lunshen‐120 approved by the Ethics Committee on Clinical Trial of the Third Affiliated Hospital of Southern Medical University. The subjects were adults aged 16–43 years, and none were hospitalized for a muscle‐specific disease or problem. Collection times were determined solely by surgical schedules, not experimental design. We recorded the local clock time for each sample and categorized them into two phases: morning (9:00–13:30 local time, *n* = 3; 2 male, 1 female) and evening (17:00–20:00 local time, *n* = 3; 2 male, 1 female). All samples were flash‐frozen in liquid nitrogen intraoperatively and stored at −80°C. More detailed subject information is provided in Table S1.

Goats ( *Capra hircus* , 3‐month‐old males) were purchased from Chengdu Dossy Experimental Animal CO. LTD, China, and were maintained under a 12:12 LD cycle. Tibialis anterior (TA) muscles were collected at ZT0 (8:00 local time) and ZT12 (20:00 local time) and stored at −80°C until further analysis.

Pigs ( *Sus scrofa* , ~ 100 kg, 3‐month‐old males) were maintained under natural light conditions at a local farm in Chongqing, China. Longissimus dorsi muscles were collected in the morning (corresponding to 6:00–8:00 local time) and evening (18:00–20:00 local time) and stored at −80°C until further analysis.

Zebrafish ( *Danio rerio* , 2‐month‐old males) were maintained on a 14‐h light/10‐h dark cycle at the Soochow University Zebrafish Facility according to standard protocols. *Clocka* knockout zebrafish (*clocka* ^*−/−*^) were generated through TALEN methods by microinjection into one‐cell zebrafish embryos. Muscle samples of Wild‐type AB strain and *clocka* ^*−/−*^ fish were collected every 4 h continuously for 24 h in DD (CT0–CT20, corresponding to 8:00–4:00 [the next day] local time).

Samples from *Aplodinotus grunniens* , *Oreochromis niloticus* , *Eriocheir sinensis* , *Procambarus clarkii* and *Macrobrachium rosenbergii* (2‐month‐old males) were collected by authors from the Freshwater Fisheries Research Center of the Chinese Academy of Fishery Sciences, Wuxi, China. For *Aplodinotus grunniens* , samples were collected at ZT0, 4, 8, 12, 16 and 20, corresponding to 8:00, 12:00, 16:00, 20:00, 0:00 and 4:00 (the next day) local time, respectively. For *Oreochromis niloticus* , *Eriocheir sinensis* , *Procambarus clarkii* and *Macrobrachium rosenbergii* (2‐month‐old males), samples were collected at ZT0, ZT6, ZT12 and ZT18, corresponding to 8:00, 14:00, 20:00 and 2:00 (the next day) local time, respectively. Samples from *Cyprinus carpio* and *Carassius auratus* (2‐month‐old males) were obtained by authors from the research base of the Freshwater Fisheries Research Center of the Chinese Academy of Fishery Sciences located at the Honghe Hani terrace of Yuanyang City, Yunnan Province, China. These samples were collected at ZT0, ZT6, ZT12 and ZT18, corresponding to 8:00, 14:00, 20:00 and 2:00 (the next day) local time, respectively. Samples from *Litopenaeus vannamei* were obtained at a local farm in Maoming City, Guangdong Province, China. These samples were collected at ZT0 and ZT12, corresponding to 8:00 and 20:00 local time, respectively.

Wild‐type *w* ^*1118*^, *per* mutant (*per* ^*0*^) and *tim* mutant (*tim* ^*0*^) fruit fly *Drosophila melanogaster* strains were purchased from Bloomington Drosophila Stock Center (Bloomington, IN, USA). Whole thorax samples were collected for the subsequent detection of *myoglianin* expression.

The wild‐type *Caenorhabditis elegans* ( *C. elegans* ) strain N2 was obtained from the *Caenorhabditis* Genetics Center (University of Minnesota, USA) and routinely maintained at 20°C on solid nematode growth medium (NGM) seeded with *Escherichia coli* OP50 as a food source. For synchronization, gravid adult N2 worms were collected and subjected to bleaching using a freshly prepared solution consisting of 1 part 5 N NaOH, 2 parts 5% sodium hypochlorite and 3 parts distilled H₂O. The mixture was incubated with gentle agitation for 3–5 min until the adult carcasses were dissolved, and the released eggs were washed three times with M9 buffer to remove residual bleach. Cleaned eggs were transferred to unseeded NGM plates and incubated overnight at 20°C to allow hatching and obtain synchronized, starved L1 larvae. L1 larvae were either harvested immediately (0 h) or transferred to fresh NGM plates seeded with OP50 and maintained at 20°C to develop into later larval stages. ZT points were defined as follows: ZT1, 5, 9 and 13 correspond to 9:00, 13:00, 17:00 and 21:00 local time, respectively.

### 2.3 Body Composition Measurement

Body composition, including total body fat, lean mass and fluid content, was evaluated in living animals without anaesthesia using a small animal Whole Body Composition Analyser (Minispec LF50, Bruker, Billerica, MA, USA). Each mouse was placed in a specially designed plastic holder, ensuring restraint without sedation or anaesthesia, and then positioned within the MRI system's measuring space. To achieve accurate measurements, the mice were immobilized within the holder during the scan, which took approximately 2 min per animal.

### 2.4 Calorimetric Assessment

An indirect calorimetry system (Oxymax, Columbus Instruments, Columbus, OH, USA) was employed to measure the day and night oxygen consumption (VO_2_) and carbon dioxide production (VCO_2_) of the mice. The system was housed within the animal facility at CAM‐SU, where environmental conditions were strictly controlled: temperature was maintained at 24°C, and the light–dark cycle was set to 12 h light (8:00–20:00) and 12 h dark (20:00–8:00 [the next day]). Each mouse was placed in a chamber with free access to food and water. The experiment spanned 3 days, with the first day designated for the mice to acclimate to the chambers. Data were corrected for energy expenditure relative to each mouse's body weight, and average energy expenditures during the day (8:00–20:00) and night (20:00–8:00 [the next day]) were calculated as the mean values of all recorded data points.

### 2.5 Treadmill Running Performance Assessment

Mice were acclimated to treadmill running over a 5‐day period, during which they ran at a speed of 5 m/min for 5 min each day. For the experimental trials, the treadmill was set at a 15% incline with an electric shock stimulus of 0.7 mA to encourage running. On the day of the experiment, the treadmill and indirect calorimetry programs were run simultaneously. The treadmill protocol began at 5 m/min for 5 min, with the speed increasing by 2.5 m/min every 2 min, eventually reaching a final speed of 25 m/min, which was maintained for 4 min. The total running duration was 25 min. All treadmill running performance assessments were conducted in the afternoon, between ZT6 and ZT8 (14:00–16:00 local time). Following the treadmill running, the mice were removed, and the treadmill was cleaned with 75% ethanol.

### 2.6 Total RNA Extraction and Real‐Time PCR

Total RNA was extracted from cells or tissues using Trizol Reagent (Life technologies), following the manufacturer's instructions. The purity and concentration of RNA were determined using a spectrophotometer (Nanodrop 3000, Thermo Fisher Scientific) by measuring absorbance at 260 and 280 nm. All samples had 260/280 nm absorption ratios close to 2, indicating high RNA purity. The RNA was then reverse transcribed into cDNA using random primers and M‐MLV reverse transcriptase. Real‐time PCR was conducted using a Roche Lightcycler 480 PCR System, with SYBR Green Master Mix (Vazyme) and gene‐specific primers either retrieved from PrimerBank or designed based on sequences from the NCBI database. Relative changes in gene expression were analysed using the 2^−ΔΔCT^ method, with mouse β‐Actin serving as the internal control.

### 2.7 Haematoxylin–Eosin (H&E) Staining

Skeletal muscle tissues from *Bmal1* ^*MKO*^ and control littermate mice were dissected and embedded in a thin layer of Tissue‐Tek O.C.T. compound (Sakura). The tissues were rapidly snap‐frozen in liquid nitrogen‐cooled isopentane and stored at −80°C until further processing. Frozen muscle tissues were sectioned into continuous 10‐μm‐thick cross‐sections using a Leica EG1150H embedding machine. For H&E staining, the sections were allowed to air‐dry overnight at room temperature. The sections were then rehydrated in phosphate‐buffered saline (PBS) for 5 min and stained with haematoxylin for 1 min to visualize nuclei. Excess haematoxylin was rinsed off under running tap water for 2 min, followed by a brief immersion in a clarifier to remove non‐nuclear staining. The sections were then stained with eosin for 10 s to visualize the cytoplasm and connective tissue. After dehydration in a series of graded ethanol and clearing in xylene, the slides were mounted with neutral resin and observed under an OLYMPUS IX73 microscope.

### 2.8 Glucose Tolerance Test (GTT)

Mice were fasted for 14 h overnight before receiving an intraperitoneal injection of 200 mg/mL D‐glucose, diluted in saline (2 g/kg body weight for mice on a chow diet). Blood glucose levels were measured from tail vein samples at 15‐, 30‐, 60‐, and 120‐min post‐injection using a glucometer (Accu‐Check Active, Roche). During the GTT, mice were randomly assigned to cages in a blinded manner to ensure unbiased results.

### 2.9 Western Blots

Proteins were extracted from homogenized muscle samples using RIPA buffer (Beyotime) supplemented with a protease inhibitor cocktail (Selleck) and phosphatase inhibitors NaF and Na_3_VO_4_. Protein concentrations were determined using the Pierce BCA Protein Assay Kit (Thermo Fisher Scientific). Equal amounts of protein were separated by SDS‐PAGE and transferred onto a polyvinylidene fluoride (PVDF) membrane (Millipore Corporation). The membranes were blocked with 5% fat‐free milk in TBST buffer for 1 h at room temperature. Primary antibodies were then incubated with the membranes overnight at 4°C. The primary antibodies used in this work include Anti‐MSTN (19 142–1‐AP, Proteintech), Anti‐BMAL1 (14 268–1‐AP, Proteintech), Anti‐HSP90 (4874S, CST), Anti‐β‐Tubulin (2146S, CST), Anti‐AKT (9272S, CST), Anti‐Phospho‐Akt (9271S, CST) and Anti‐FLAG (F1804, Sigma). The following day, membranes were incubated with an HRP‐conjugated secondary antibody (Jackson ImmunoResearch) for 1 h at room temperature. Signal detection was carried out using an enhanced chemiluminescence (ECL) substrate (Vazyme), with signals visualized on a chemiluminescence imaging system (ChemiScope 6100 Touch). For all analyses, mRNA and protein samples were obtained from the same individuals unless otherwise specified.

### 2.10 Statistical Analysis

The rhythmicity of gene expression was evaluated using JTK analysis, with a *p*‐value of < 0.05 considered indicative of rhythmicity. Data are presented as mean ± SEM. Statistical significance was determined using an unpaired *t*‐test or two‐way ANOVA for multiple comparisons, with significance levels indicated as follows: *p < 0.05, **p < 0.01, ***p < 0.001, ****p < 0.0001.

## 3 Results

### 3.1 Myostatin Is Rhythmically Expressed in the Mouse Skeletal Muscle

**FIGURE 1:**
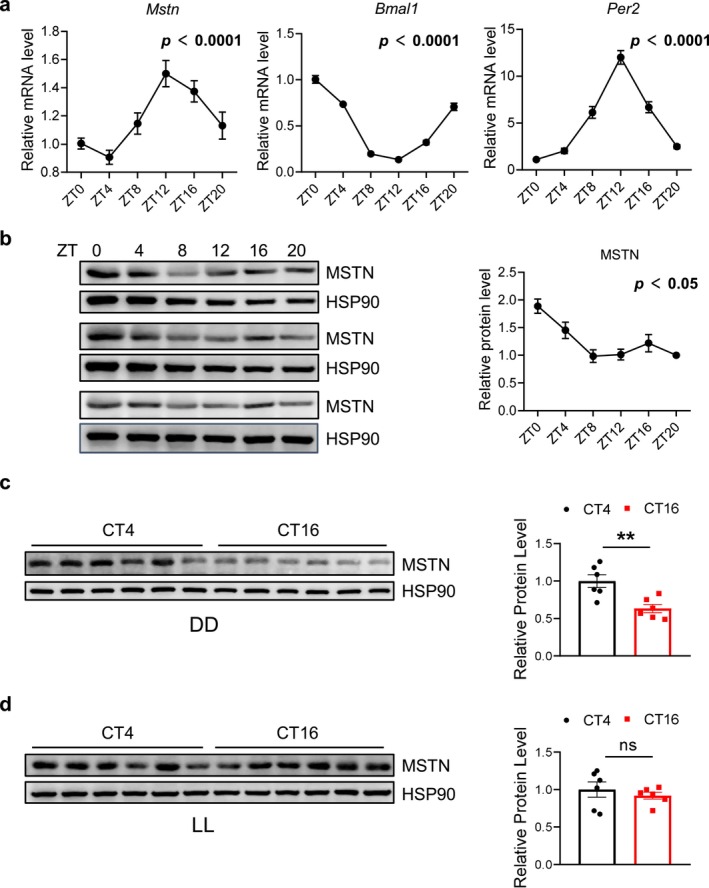
*Mstn* exhibits rhythmic expression in mouse skeletal muscle. (a) The mRNA expression patterns of *Mstn*, *Bmal1* and *Per2* at different time points in mouse skeletal muscle (*n* = 5–7/time point). (b) The protein expression patterns and quantitative analysis of MSTN at different time points in mouse skeletal muscle (*n* = 5/time point). (c) The protein expression of MSTN in mouse skeletal muscle at CT4 (12:00 local time) and CT16 (24:00 local time) under DD conditions (*n* = 6/group). (d) The protein expression levels of MSTN in mouse skeletal muscle at CT4 (12:00 local time) and CT16 (24:00 local time) under LL conditions (*n* = 5/group). The rhythmicity of gene expression was assessed using JTK analysis, with *p* < 0.05 considered rhythmic. Data presented as mean ± SEM, analysed by unpaired *t*‐test, **p < 0.01, ns = not significant.

We examined gene expression of myostatin and clock‐related genes in Anterior tibial (TA) muscle from wildtype (WT) mice every 4 h for consecutive 24 h. qRT‐PCR results indicated that key circadian clock genes robustly oscillated in TA muscle, including *Bmal1*, *Clock*, *Per1*, *Per2*, *Per3*, *Cry1*, *Cry2*, *Rev‐erba* and *Rev‐erbb* (Figures 1a and S1). The mRNA level of *Myostatin* (*Mstn*) also significantly oscillated in TA muscle and peaks at ZT 12 (20:00 local time), displaying a similar pattern with *Per2*, but anti‐phasic to *Bmal1* (Figure 1a). Protein levels of MSTN also showed significant oscillations in TA muscle, peaking at ZT 0 (8:00 local time) (Figure 1b). To exclude the potential effect of light on MSTN expression, we placed the mice in DD for 2 weeks before collecting their muscle at ZT 0 (8:00 local time) and ZT 12 (20:00 local time), respectively. Western blot results indicated that MSTN protein also shows diurnal variability of MSTN under DD (Figure 1c). In addition, we also placed WT mice in LL to disrupt their circadian rhythm (Figure S2). After 4 weeks of LL treatment, mice show significant arhythmicity in wheel running and the diurnal variability of MSTN protein expression disappeared (Figures 1d and S2). These results demonstrate that *Mstn* is rhythmically expressed in the mouse skeletal.

### 3.2 Rhythmicity of Myostatin Expression in Muscle Is Evolutionarily Conserved From *C. elegans* to Human and Is Regulated by the Circadian Clock

**FIGURE 2:**
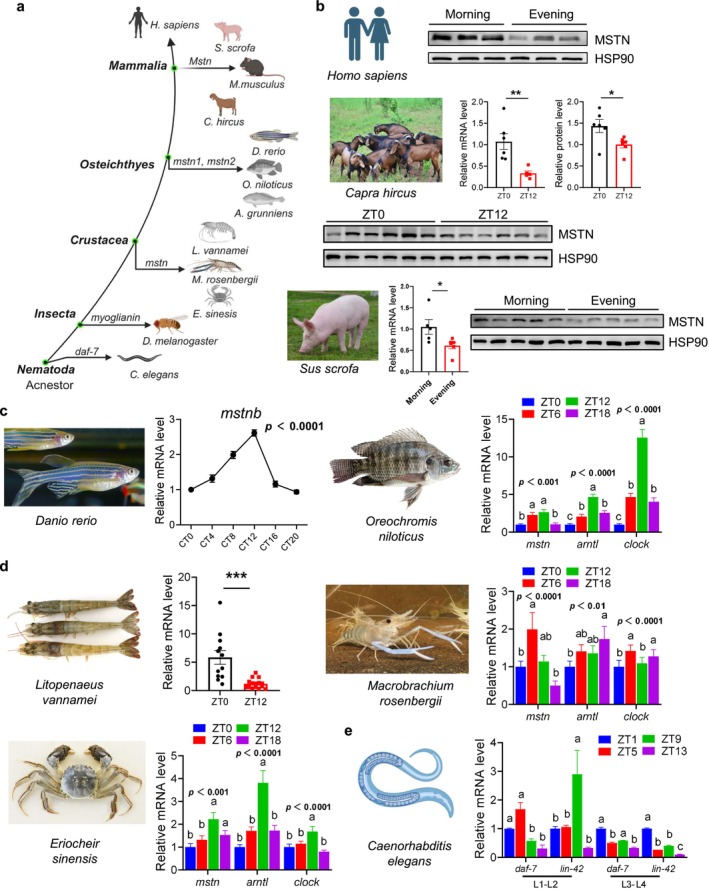
The rhythmicity of *Mstn* expression in muscle is conserved across species. (a) *Mstn* orthologs during evolution. (b) Mammals: The protein expression of MSTN in human skeletal muscle in the morning and evening (*n* = 3/group). The mRNA and protein expression of Mstn in *Capra hircus* skeletal muscle at ZT0 (8:00 local time) and ZT12 (20:00 local time) (*n* = 6/group). The mRNA and protein expression of Mstn in *Sus scrofa* skeletal muscle in the morning and evening (*n* = 5/group). (c) Fish: The mRNA expression pattern of *mstnb* in *Danio rerio* skeletal muscle at different time points (*n* = 3/time point). The mRNA expression of *mstn*, *arntl* and *clock* in *Oreochromis niloticus* skeletal muscle at different time points (*n* = 14–18/time point). (d) Crustacean: The mRNA expression of *mstn* in *Litopenaeus vannamei* muscle tissue at ZT0 (8:00 local time) and ZT12 (20:00 local time) (*n* = 12–15/group). The mRNA expression of *mstn*, *arntl* and *clock* in *Macrobrachium rosenbergii* muscle tissue at different time points (*n* = 8–11/time point). The mRNA expression of *mstn*, *arntl* and *clock* in *Eriocheir sinensis* muscle tissue at different time points (*n* = 11–16/time point). (e) The mRNA Expression of *daf‐7* and *lin‐42* in *Caenorhabditis elegans* at the L1 and L2 and L3 and L4 stages at different time points (*n* = 2/time point). Data presented as mean ± SEM, analysed by unpaired *t*‐test with Bonferroni correction, bars sharing the same lowercase letter indicate no significant difference (*p* > 0.05). *p < 0.05, **p < 0.01, ***p < 0.001.

Muscle represents one of the most evolutionarily conserved motor tissues, originating from primitive bilateral symmetry animals, such as *C. elegans* [s1]. Sequence analysis revealed that MSTN (GDF‐8) and GDF‐11 shared the same ancestor DAF‐7 in *C. elegans* . MSTN and GDF‐11 diverged at the time of the emergence of vertebrates (Figure 2a). The *mstn* gene is uniquely duplicated in *mstna* and *mstnb* in fish (Figure 2a). As the circadian clock is evolutionarily conserved in the animal kingdom, including *C. elegans* , we investigate whether the rhythmicity of *Mstn* in muscle expression is also ubiquitous across different species. We collected muscle samples at different times from human, pig, goat, fish, shrimp, crab, and worms to examine *Mstn* expression. MSTN in skeletal muscles of all mammals examined, including *Homo sapiens* , *Capra hircus* and *Sus scrofa* , displays significant diurnal variability at protein levels, with being higher in the morning and lower in the evening (Figure 2b).In addition, mRNA levels of *C. hircus Mstn* also show significantly higher in the morning and lower in the evening (Figure 2b). In zebrafish ( *Danio rerio* ), the mRNA level of *mstnb* showed significant oscillations under DD condition (Figure 2c). In other fishes including *Oreochromis niloticus* and *Cyprinus carpio*, mRNA levels of *mstn1* in muscles also showed significant oscillations (Figure 2c, S3a); *Carassius auratus* displayed distinct diurnal variation of *mstn1* expression (Figure S3b). Skeletal muscle commonly constitutes the largest tissue mass in the invertebrate *Crustacea*. The mRNA level of *mstn* was significantly higher in the morning and lower at night in the muscles of shrimp *Litopenaeus vannamei* (Figure 2d). Similarly, *Macrobrachium rosenbergii* and *Eriocheir sinesis mstn* mRNA expressions oscillated in the muscles (Figure 2d), *Procambarus clarkia* displayed distinct diurnal variation of *mstn* expression (Figure S3c). In the ancient *C. elegans* , the homologue of the *Per* gene *lin‐42* has been shown to oscillate during the early development of the worm at L1–L4. Thus, we collected time‐course samples during L1–L4, and the results indicated that the expression levels of *daf‐7* varied at different times of the day during development and shared a similar expression pattern with Lin‐42 at the L3–L4 stage (Figure 2e). Together, the expression profiles of *Mstn* in different species indicate that its rhythmicity is evolutionarily conserved in muscles.

### 3.3 *Bmal1* Deletion Downregulates *Mstn* and Results in Larger Skeletal Muscle Mass

**FIGURE 3:**
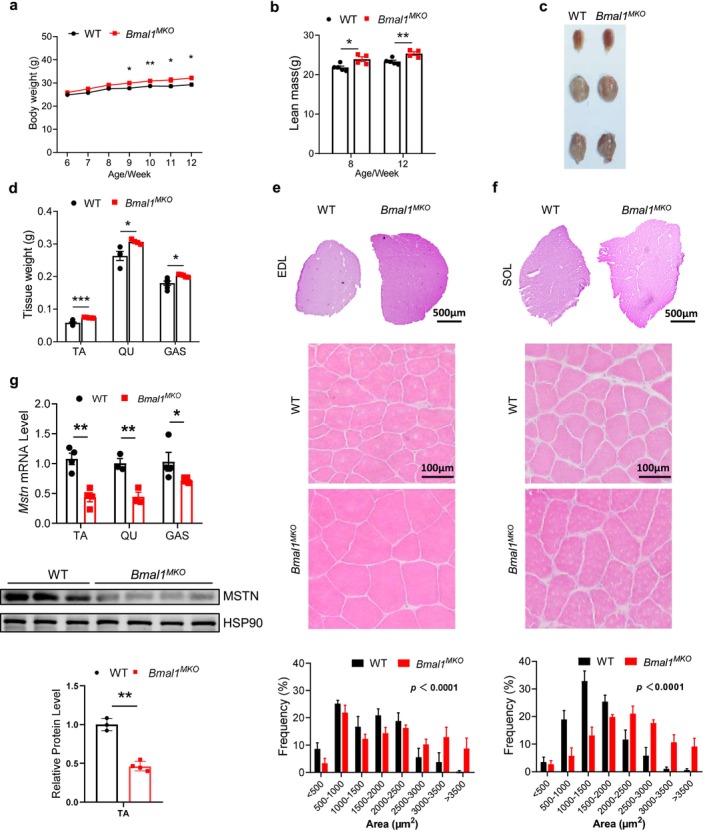
Loss of *Bmal1* increases skeletal muscle mass and reduces muscle MSTN levels in mice. (a) Body weight curves of *Bmal1* ^*MKO*^ mice and control littermates from 6 to 12 weeks of age (*n* = 4–5/group). (b) Lean mass of *Bmal1* ^*MKO*^ mice and control littermates at 8 and 12 weeks of age (*n* = 4–5/group). (c) Representative images and weights of TA, QU and GAS muscle tissues of dissected *Bmal1* ^*MKO*^ mice and control littermates at 12 weeks of age. (d) Weights of TA, QU and GAS skeletal muscle tissues of dissected *Bmal1* ^*MKO*^ mice and control littermates at 12 weeks of age (*n* = 4–5/group). (e) HE‐stained morphology images of EDL tissue frozen sections of *Bmal1* ^*MKO*^ mice and control littermates. Scale bar: 500 μm in upper panels (overview), 100 μm in lower panels (magnified views). Cross‐sectional area distribution of EDL tissue of *Bmal1* ^*MKO*^ mice and control littermates (*n* = 4–5/group). (f) HE‐stained morphology images of SOL tissue frozen sections of *Bmal1* ^*MKO*^ mice and control littermates. Scale bar: 100 μm. Cross‐sectional area distribution of SOL tissue from *Bmal1* ^*MKO*^ mice and control littermates (*n* = 4–5/group). (g) Relative mRNA levels of *Mstn* in TA, Qu and GAS muscles from *Bmal1* ^*MKO*^ mice and control littermates. Protein level and quantification of MSTN in TA muscle of *Bmal1* ^*MKO*^ mice and control littermates (*n* = 3–4/group). Data presented as mean ± SEM, analysed by unpaired *t*‐test or two‐way ANOVA for multiple comparisons, *p* < 0.0001 for genotype effect. *p < 0.05, **p < 0.01, ***p < 0.001.

We next attempt to identify the potential regulator of *Mstn*. BMAL1, as the master transcriptional regulator of the circadian clock, plays the key role in the skeletal muscle clock [s2]. Thus, we determined the protein level of BMAL1 at different time points of the day and found it peaked at ZT0 (8:00 local time) (Figure S4a), displaying an antiphasic pattern to *Mstn* mRNA (Figure 1a). Then, we crossed *Bmal* ^*fl/fl*^ mice with *Ckmm‐cre* to generate the muscle‐specific *Bmal1* knockout mouse model (*Bmal1* ^*MKO*^). Notably, we observed a significant increase in total body weight of *Bmal1* ^*MKO*^ mice at 9–12 weeks of age (Figure 3a) and a significant increase in lean mass at 8 and 12 weeks of age (Figure 3b), while the difference in lean mass at 12 weeks of age (~ 1.996 g) closely matches the difference in total body weight (~ 2.000 g). Consistent with previous studies using muscle‐specific *Bmal1* knockout models with early developmental deletion [s3, s4], we observed increased muscle mass including TA, QU (quadriceps) and GAS (gastrocnemius) and fibre size in *Bmal1* ^*MKO*^ mice (Figure 3c–f). However, as lean mass includes tissues other than skeletal muscle, it is unlikely that the augmentation in skeletal muscle mass alone accounts for this difference. Additionally, since the *Ckmm‐Cre* model is also expressed in the heart, we further assessed heart weight and cardiac function of WT and *Bmal1* ^*MKO*^ mice. We observed a slight but not significant increase in heart weight and no significant changes in cardiac function (Figure S5).

We then conducted RNA‐seq analysis to identify the differentially expressed genes (DEGs) in *Bmal1* ^*MKO*^ mice (Table S2). A total of 1986 DEGs were identified, with 1035 upregulated DEGs and 951 downregulated DEGs (Figure S4b). The expression levels of key circadian clock genes, including *Clock*, *Pers*, *Crys*, *Dbp*, *Nr1d1* and *Rora*, were all significantly altered in TA of *Bmal1* ^*MKO*^ mice (Figure S4c). GO analysis revealed that genes involved in the TGF‐β receptor signalling pathway were significantly enriched in the downregulated genes (Figure S4d), in which we found that the expression level of *Mstn* was significantly decreased after *Bmal1* deletion (Figure S4e). qRT‐PCR and western blotting analyses confirmed that both mRNA and protein levels of *Mstn*/MSTN were down‐regulated in muscles of *Bmal1* ^*MKO*^ mice compared to those of WT (Figure 3g). MSTN controls skeletal muscle mass through SMAD‐pAKT signalling pathway [s5]. Consistently, we found that both levels of AKT and pAKT were increased after *Bmal1* knockout, while the pAKT/AKT ratio showed a modest elevation that was not statistically significant (Figure S6a,b).

In the glucose tolerance test, *Bmal1* ^*MKO*^ mice showed increased glucose levels after intraperitoneal (i.p.) injection of excess glucose (Figure S7a). No difference in the oxygen consumption (VO_2_), carbon dioxide production (VCO_2_) or respiration exchange rate (RER) was detected between WT and *Bmal1* ^*MKO*^ mice (Figure S7d–f). In addition, VO_2_, VCO_2_, RER and maximum running distance of the mice during the treadmill were not changed (Figure S7b,g,h). These data together indicate that the loss of *Bmal1* in skeletal muscle leads to greater muscle mass in mice.

### 3.4 Rhythmicity of Myostatin Expression in Muscle Is Controlled by the Conserved Circadian Clock

**FIGURE 4:**
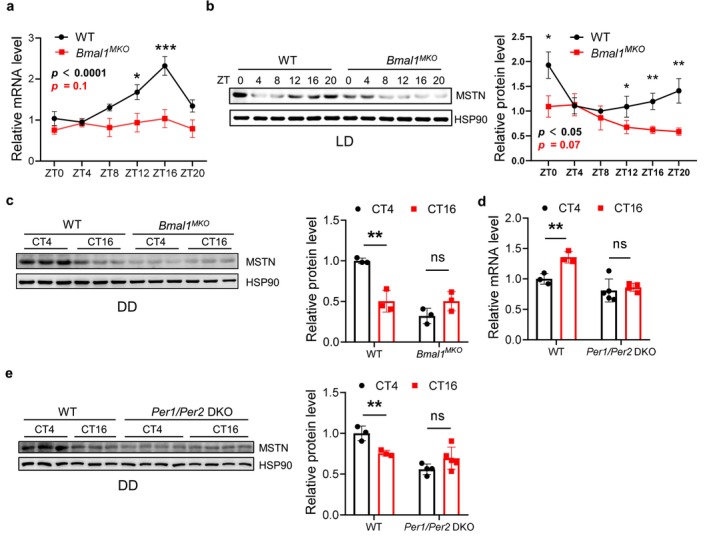
The rhythmic expression of MSTN is regulated by internal biological clock. (a) The mRNA expression pattern of *Mstn* at different time points in the TA tissues of *Bmal1* ^*MKO*^ mice and control littermates (WT, *n* = 4–5/time point; *Bmal1* ^*MKO*^, *n* = 4–5/time point). (b) The protein expression pattern and grayscale quantification of MSTN at different time points in the TA tissues of *Bmal1* ^*MKO*^ mice and control littermates (WT, *n* = 4/time point; *Bmal1* ^*MKO*^, *n* = 4/time point). (c) The protein expression and grayscale quantification of MSTN at CT4 (12:00 local time) and CT16 (24:00 local time) in the TA tissues of *Bmal1* ^*MKO*^ mice and control littermates under DD conditions. (WT, *n* = 3/group; *Bmal1* ^*MKO*^, *n* = 3/group). (d) The mRNA expression of *Mstn* at CT4 (12:00 local time) and CT16 (24:00 local time) in the TA tissues of *Per1/Per2* DKO mice and control littermates under DD conditions (WT, *n* = 3/group; DKO, *n* = 4–5/group). (e) The protein expression and grayscale quantification of MSTN at CT4 (12:00 local time) and CT16 (24:00 local time) in the TA tissues of *Per1/Per2* DKO mice and control littermates under DD conditions (WT, *n* = 3/group; DKO, *n* = 4/group). The rhythmicity of gene expression was assessed using JTK analysis, with *p* < 0.05 considered rhythmic. Data presented as mean ± SEM, analysed by unpaired *t*‐test, *p < 0.05, **p < 0.01, ***p < 0.001, ns = not significant.

We then investigated how the circadian clock regulates the rhythmic expression of *Mstn*. We collected TA muscles from WT and *Bmal1* ^*MKO*^ mice at different time points of the day. qRT‐PCR results showed that the oscillation of *Mstn* disappeared (Figure 4a) in *Bmal1* ^*MKO*^ mice. In addition, the expression levels of *Mstn* were significantly reduced at ZT12 (20:00 local time), ZT16 (24:00 local time) and ZT20 (4:00 [the next day] local time) (Figure 4a). Similar results were also observed from the protein expression of MSTN, which showed loss of rhythmic expression and were significantly decreased at ZT0 (8:00 local time), ZT16 (24:00 local time) and ZT20 (4:00 [the next day] local time) (Figure 4b). Consistently, protein levels of AKT and pAKT at different times of the day were upregulated (Figure S6c). To assess whether feeding behaviour might contribute to the observed rhythmic expression of *Mstn*, we monitored food intake in wild‐type and *Bmal1* ^*MKO*^ mice at 4‐h intervals over a 48‐h period. In wild‐type mice, food intake displayed a clear diurnal oscillation that corresponded with changes in MSTN mRNA levels (Figure S7c). In contrast, *Bmal1* ^*MKO*^ mice exhibited no significant differences in the pattern or quantity of food intake compared to wild‐type controls, despite the loss of *Mstn* rhythmicity (Figure 4a, S7c). These results suggest that while feeding behaviour may be associated with *Mstn* expression in wild‐type mice, the disruption of *Mstn* rhythmicity in *Bmal1* ^*MKO*^ mice is not attributable to altered feeding patterns. We then confirmed the results by placing the mice in DD. Western‐blot results showed that the diurnal variability of MSTN protein expression disappeared in TA of *Bmal1* ^*MKO*^ compared to those of WT mice (Figure 4c). In addition, the MSTN protein level was significantly decreased at CT4 (12:00 local time) (Figure 4c).

BMAL1 binds to the E‐box region of clock‐control genes to regulate their transcription [s6], while *Mstn* has been reported to be transcriptionally activated by myogenic factors through direct E‐box binding [s7]. We next examined whether BMAL1 regulated *Mstn* transcription through direct E‐box binding. Sequence analysis of the 2‐kb *Mstn* promoter region revealed 7 E‐box elements (Figure S8a). We then performed ChIP assays using anti‐BMAL1 antibody on TA muscles collected at ZT4 (12:00 local time) and ZT16 (24:00 local time) and ran ChIP‐qPCR to test the binding of BMAL1 to the E‐box regions on the *Mstn* promoter. From ChIP‐seq, we did not detect any potential BMAL1 binding peaks on the *Mstn* genome region (Table S3). A similar result was also observed from ChIP‐qPCR, in which BMAL1 showed no binding to any of the *Mstn* E‐box regions, but positive binding to the E‐box region of *Per1* (Figure S8b). In addition to the E‐box, the D‐box regulated by DBP is also a key element in the control of circadian gene transcription [s8]. Intriguingly, we confirmed the binding of BMAL1 to the *Dbp* genome region and found that loss of *Bmal1* down‐regulates *Dbp1* (Tables S2 and S3). We next examined if BMAL1 regulates *Mstn* transcription through DBP and the D‐box. We were able to identify 2 potential D‐box motifs (TGATGCAA, TTACTCAA, [s9]) in the promoter region of *Mstn* (Figure S9a). Due to a lack of commercially available DBP antibody for ChIP, we constructed a stable *Dbp*‐overexpressing C2C12 cell line and performed ChIP‐qPCR (Figure S9b). The result indicated that DBP binds to the D‐box region of *Per2* but not *Mstn*. Therefore, although *Bmal1‐KO* abolishes the circadian pattern of *Mstn*, it does not transcriptionally regulate *Mstn*. To assure that *Mstn* expression is directly regulated by the circadian clock but not indirectly influenced by muscle mass change due to *Bmal1* knockout, we constructed another circadian mutant mouse model, in which *Per1* and *Per2* were both knocked out (*Per1/Per2* DKO) [s10]. While TA muscles of WT mice showed significantly higher *Mstn* expression at CT16 (24:00 local time) compared to CT4 (12:00 local time) under DD, the *Per1/Per2* DKO muscles had similar levels of *Mstn* at CT16 (24:00 local time) and CT4 (12:00 local time) (Figure 4d). Consistently, protein levels of MSTN were significantly higher at CT4 (12:00 local time) compared to CT16 (24:00 local time) in WT mice, but no differences were detected in the *Per1/Per2* DKO mice (Figure 4e). These data collectively demonstrate that rhythmic expression of *Mstn* in mouse skeletal muscle is controlled by the circadian clock.

To further investigate potential indirect mechanisms by which the circadian clock may regulate *Mstn* expression, we analyzed the RNA‐seq results from skeletal muscle of *Bmal1* ^*MKO*^ and control mice. We found that the mRNA expression of the histone acetyltransferases *Kat14* and *Kat5* was significantly downregulated, while the mRNA expression of the histone deacetylases *Hdac11* and *Sirt5* was significantly upregulated in the skeletal muscle of *Bmal1* ^*MKO*^ mice (Figure S10a). Subsequently, we validated the mRNA expression of *Kat14* and *Kat5* in skeletal muscles collected from *Bmal1* ^*MKO*^ and control mice at different time points (Figure S10b). Given the role of histone acetylation in gene transcription, we next assessed the acetylation level of histone H3 at lysine 27 (H3K27ac), a marker of active chromatin. Western blot analysis revealed that H3K27ac levels were significantly decreased in *Bmal1* ^*MKO*^ muscle, and its normal rhythmic pattern was disrupted (Figure S10c). Similar reductions and loss of rhythmicity in H3K27ac were also observed in *Per1/Per2* DKO muscle (Figure S10c). These results suggest that circadian disruption may alter the epigenetic landscape in skeletal muscle, potentially influencing *Mstn* transcription. However, the precise molecular mechanisms remain unclear.

**FIGURE 5:**
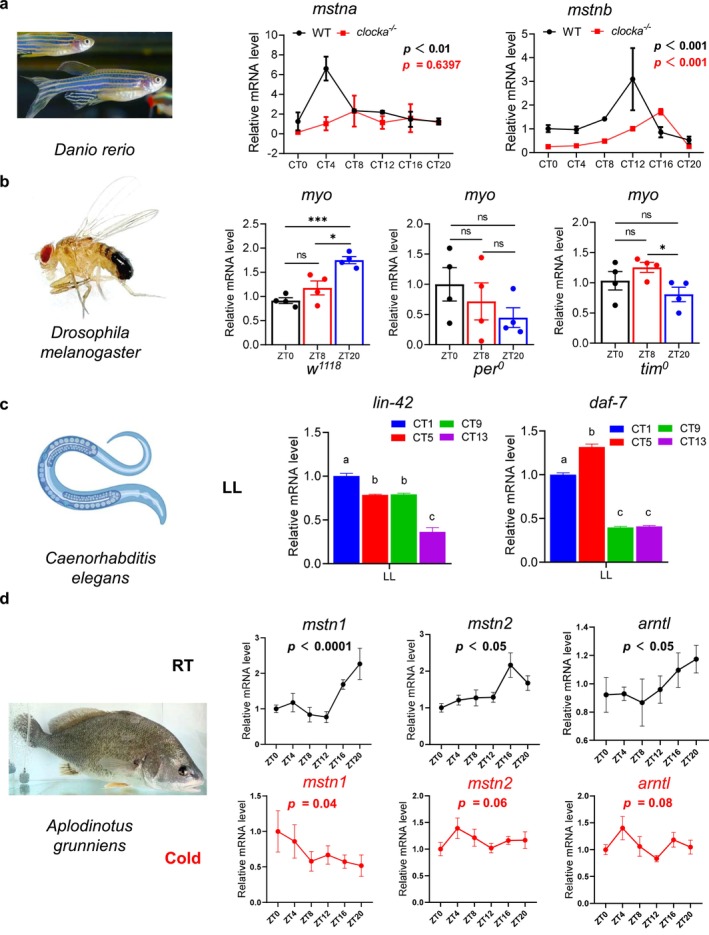
The regulatory mechanism of rhythmic *Mstn* expression is conserved across species. (a) The mRNA expression patterns of *mstna* and *mstnb* in the skeletal muscle tissue of *clocka* ^*−/−*^ and control *Danio rerio* at different time points (WT, *n* = 3/time point; *clocka* ^*−/−*^, *n* = 3/time point). (b) The mRNA expression patterns of *mstn* in *w* ^*1118*^, *per* ^*0*^ and *tim* ^*0*^ *Drosophila melanogaster* at different time points (*w* ^*1118*^, *per* ^*0*^ and *tim* ^*0*^, *n* = 4/time point). (c) The mRNA expression patterns of *daf‐7* and *lin‐42* in *Caenorhabditis elegans* at different time points under LL conditions (*n* = 3/time point). (d) The mRNA expression patterns of *mstn1*, *mstn2* and *arntl1* in the skeletal muscle of *Aplodinotus grunniens* at different time points under RT and cold conditions (RT, *n* = 7–12/time point; cold, *n* = 9–12/time point). The rhythmicity of gene expression was assessed using JTK analysis, with *p* < 0.05 considered rhythmic. Data presented as mean ± SEM, analysed by unpaired *t*‐test with Bonferroni correction, bars sharing the same lowercase letter indicate no significant difference (*p* > 0.05). *p < 0.05, ***p < 0.001, ns = not significant.

We next investigated whether the circadian clock‐mediated regulatory mechanism of *Mstn* rhythmicity is conserved across different species. The zebrafish circadian locomotor output cycles kaput a (*clocka*) gene encodes a core component of the circadian clock that regulates daily rhythms of gene expression and physiological processes. In *clocka*‐mutant zebrafish (*clocka* ^*−/−*^), loss of functional *clocka* gene led to loss of behavioural rhythmicity at DD [s11]. The mRNA levels of *D. rerio mstna* lost their oscillation and were significantly decreased in *clocka* ^*−/−*^ fish under DD, while *mstnb* preserved the rhythmic expression but had reduced amplitude of oscillation (Figure 5a).

We next collected the thorax at 8:00, 16:00, and 4:00 (the next day) local time. From wild‐type (*w* ^*1118*^), *per* ^*0*^ and *tim* ^*0*^ fruit flies, we examined the expression levels of *Drosophila melanogaster myoglianin (GDF11)*. The results showed that *myo* exhibited significant diurnal variability at *w* ^*1118*^ flies (Figure 5b). In *per* ^*0*^ and *tim* ^*0*^ lines, this robust diurnal pattern was disrupted; however, *tim* ^*0*^ flies still exhibited a residual rhythmic or patterned expression, albeit with altered phase compared to wild‐type (Figure 5b).

Interestingly, both *C. elegans lin‐42* and *daf‐7* preserved their diurnal variability during L3–L4 under LL conditions (Figure 5c). *Aplodinotus grunniens* is a freshwater fish that is hypothermia tolerant with reduced activity and food intake at lower temperatures. We found that compared to those fish at normal temperature (22°C), oscillations of circadian gene *arntl* mRNA expression were disrupted at 6°C (Figure 5d). Notably, despite the lack of *arntl* oscillation, *mstn1* expression remained statistically rhythmic under cold conditions (*p* = 0.04) but with changes in pattern, while *mstn2* mRNA oscillation was abolished. These data collectively indicated that circadian regulation of rhythmic *Mstn* expression in muscles is highly conserved across different species.

## 4 Discussion

Our study uncovers an evolutionarily conserved circadian regulation of *Mstn* expression in skeletal muscle—a previously unrecognized layer of complexity in MSTN biology. Since the discovery of MSTN in 1997, numerous studies have focused on the role of this new member of the TGF‐β superfamily as a conserved negative regulator of skeletal muscle mass across species [21]. Both naturally occurring and genetically manipulated mutations of *Mstn* lead to increased muscling in various species, throughout vertebrates from fish to mammals [26]. In this study, we extended the current understanding of MSTN beyond its muscle‐repressive function and demonstrated that rhythmic *Mstn* expression represents a fundamental biological feature conserved even in invertebrates.

MSTN shares evolutionary ancestry with its homologous protein GDF‐11 in invertebrates such as *C. elegans* , *D. melanogaster* , and *Crustacea* [s5]. We found that *MSTN* in these ancestors, such as *daf‐7* and *myo*, also exhibits circadian oscillations. Following the divergence of myostatin and GDF‐11 during vertebrate evolution, specialized MSTN proteins emerged. Thus, fish carry specialized MSTN proteins but have two copies of *mstn* genes. In zebrafish, *mstna* and *mstnb* are highly expressed in non‐muscle tissues, including the eyes, brain and testis, with different tissue expression patterns, but the functional significance remains to be characterized [27]. Critically, despite the high sequence similarity of *mstna* and *mstnb* [28], we observed differential circadian responses between paralogs: *clocka* ablation abolished *mstna* rhythmicity while merely dampening *mstnb* oscillation amplitude. This functional divergence correlates with augmentation in muscle mass occurring only in *mstnb* ^*−/−*^ zebrafish [29]. These findings collectively establish that circadian regulation constitutes an intrinsic property of MSTN physiology across evolutionary lineages.

The skeletal muscle circadian clock critically coordinates development, metabolism and regenerative capacity, thereby maintaining systemic homeostasis [s12]. Previous studies have reported that *Bmal1* deletion during early development causes increased skeletal muscle mass [s3, s4] but impairs systemic metabolism [30]. Consistent with these findings, there was a notable augmentation of muscle mass in our *Bmal1* ^*MKO*^ mice. Notably, the following three key observations emerge from our model: (1) augmentation in muscle mass occurred without functional improvement; (2) total body weight increased disproportionately to prior models [s3, s4], with lean mass changes suggesting contributions beyond skeletal muscle; and (3) despite Ckmm‐Cre activity in cardiac tissue, no significant cardiac enlargement or dysfunction was detected. This parallels clinical limitations of MSTN inhibition therapies, where muscle mass gains frequently fail to improve strength [31, 32]. Mechanistically, muscle mass control is achieved by both protein synthesis through pAKT‐mTOR signalling and MSTN‐mediated protein degradation [29]. Indeed, despite the downregulation of *Mstn* in *Bmal1‐null* muscle, protein levels of both AKT and pAKT are increased, raising the possibility that BMAL1 controls muscle mass through pAKT‐related protein synthesis. As a previous study has shown, BMAL1 integrates with mTOR signalling to regulate circadian protein synthesis [17]. BMAL1/CLOCK controls the circadian transcriptome through direct E‐box binding, while the key myogenic factors also recognize the E‐box region on gene promoter regions [33]. Analysis of the *Mstn* promoter region from fish to mouse reveals conserved 5 E‐box motifs in the proximal 1 kb [34]. However, ChIP‐qPCR and ChIP‐seq both revealed no direct binding of BMAL1 to any of the seven identified E‐box regions in the mouse *Mstn* promoter. DBP was also not involved in the direct transcriptional regulation of *Mstn* expression. In addition, we saw similar expression pattern changes of *Mstn* from *Bmal1* and *Per1/Per2* DKO models, which may indicate that the rhythmic expression of *Mstn* was due to the disrupted circadian rhythm, indirectly of BMAL1 and PERs. Thus, the detailed circadian regulation of *Mstn* transcription requires further investigation. Most significantly, while circadian disruption abolished *Mstn* rhythmicity across all models (*Bmal1* ^*MKO*^, *Per1/Per2* DKO, or *clocka* ^*−/−*^), baseline expression persisted—demonstrating that temporal regulation might be separable from transcriptional amplitude. This implies that loss of rhythmicity itself, rather than absolute expression levels, may drive pathological consequences.

Current therapeutic paradigms overwhelmingly focus on continuous MSTN repression for muscle‐wasting conditions (muscular dystrophies, cachexia, sarcopenia) [s13], yet completely overlook circadian dynamics. Our demonstration that rhythmic *Mstn* expression represents a fundamental biological feature conserved from invertebrates to mammals reveals a potential limitation of continuous repression strategies: They suppress overall expression but fail to preserve physiological oscillations. In mammals, MSTN exhibits pleiotropic functions beyond myoregulation, including systemic metabolic control [35], with tissue‐specific knockout models revealing compartmentalized roles (e.g., BAT‐derived vs. muscle‐derived MSTN) [36, 37, 38]. This compartmentalization is especially relevant to sarcopenia, where both muscle preservation and metabolic health are compromised in aging populations. Thus, targeted neutralization of MSTN might risk compromising MSTN's metabolic functions while perpetuating arrhythmicity. Conversely, our findings on the circadian regulation of *Mstn* expression provide a molecular basis for precision interventions. And we propose that preserving circadian integrity may yield superior outcomes. Exercise performance exhibits strong circadian dependence [39, 40], prompting our hypothesis that timed interventions could maximize its efficacy: Strategically scheduling activity during *Mstn* expression troughs might enhance skeletal muscle hypertrophy by minimizing inhibitory signaling. As our data demonstrate that the circadian regulation of MSTN crosses evolutionary lineages, this chronotherapeutic principle extends beyond clinical applications: Rhythmic *Mstn* expression in livestock suggests agricultural strategies where circadian‐aligned feeding schedules could suppress MSTN peaks to augment muscle yield. Thus, we advocate a paradigm shift from continuous repression toward rhythm‐preserving interventions—a fundamental rethinking of MSTN modulation strategies informed by circadian biology.

## Funding

This work was partially supported by the National Natural Science Foundation of China (82202654 to B.G.), the Ministry of Science and Technology (2018YFA0801102 to Y.D.), the Natural Science Foundation of Jiangsu Province (BK20230176 to C. S and BK20210715 to Z.J), the Central Public‐interest Scientific Institution Basal Research Fund, CAFS (2023TD66), the Earmarked fund for China Agriculture Research System (CARS‐48), the Natural Science Foundation of Sichuan Province (23NSFSC1804 to Y.X.), the National Key R&D Program of China (2019YFA0802400 to H.W.) and the National Natural Science Foundation of China (NSFC) (81701347 and 31961133026 to H.W.). This work was also supported by the SIP High‐Quality Innovation Platform for Chronic Diseases (YZCXPT2022203) and the Gusu Innovation and Entrepreneur Leading Talents Project (ZXL2023200 to Z.J.).

## Conflicts of Interest

The authors declare no conflicts of interest.

## Supporting information


**Figure S1:** A complete circadian clock system is present in the skeletal muscle of mice. The mRNA expression patterns of key clock genes such as *per1*, *clock*, and *cry1* at different time points in the skeletal muscle of B6J mice. The rhythmicity of gene expression was assessed using JTK analysis, with *p* < 0.05 considered rhythmic. Data presented as Mean ± SEM.
**Figure S2:** Circadian rhythms in mice are disrupted under LL conditions. Representative wheel‐running data for B6J mice under LL conditions.
**Figure S3:** The mRNA expression of mstn at different time points in muscle tissues of Cyprinus carpio, Carassius auratus and Procambarus clarkii. (a) The mRNA expression of mstn, arntl1 and clock at different time points in the muscle of Cyprinus carpio (n = 7–12/time point). (b) The mRNA expression of mstn, arntl1 and clock at different time points in the muscle of Carassius auratus (n = 5–12/time point). (c) The mRNA expression of mstn, arntl1 and clock at different time points in the muscle tissue of Procambarus clarkii (*n* = 10–18/time point). The rhythmicity of gene expression was assessed using JTK analysis, with p < 0.05 considered rhythmic. Data presented as mean ± SEM, analysed by unpaired t‐test with Bonferroni correction, bars sharing the same lowercase letter indicate no significant difference (p > 0.05).
**Figure S4:** RNA‐seq revealed a significant downregulation of Mstn mRNA expression in the skeletal muscle of Bmal1MKO mice. (a)The protein expression patterns of BMAL1 at different time points in the skeletal muscle of B6J mice. (b) RNA‐Seq volcano plot of the TA skeletal muscle in Bmal1MKO and control littermates. (c) Heatmap showing the mRNA expression of key clock genes in the TA skeletal muscle of Bmal1MKO and control littermates. (d) KEGG pathway analysis of RNA‐seq data from the TA skeletal muscle of Bmal1MKO and control littermates. (e) FPKM values of Mstn from RNA‐Seq results in the TA skeletal muscle of Bmal1MKO and control littermates. Data presented as Mean ± SEM, analysed by unpaired t‐test, **p < 0.01.
**Figure S5:** Cardiac morphology and function are comparable between Bmal1MKO and control littermates. (a) Heart weight of 12‐week‐old Bmal1MKO and control littermates. (b) Representative HE‐stained myocardial sections of Bmal1MKO and control littermates. Scale bar:50 μm. (c‐h) Echocardiographic assessment of cardiac function parameters: (c) ejection fraction (EF), (d) heart rate, (e) left ventricular internal diameter in diastole (LVIDd), (f) left ventricular internal diameter in systole (LVIDs), (g) left ventricular posterior wall thickness in diastole (LVPWd) and (h) Left ventricular posterior wall thickness in systole (LVPWs). Data presented as mean ± SEM (*n* = 4–5/group), analysed by unpaired t‐test, ns = not significant.
**Figure S6:** Bmal1MKO mice exhibit increased expression of AKT and pAKT in skeletal muscle. (a‐b) Protein expression levels and grayscale quantification of AKT, pAKT and pAKT/AKT in TA tissue of Bmal1MKO and control littermates. (c) Protein expression levels of AKT and pAKT at different time points in TA tissue of Bmal1MKO and control littermates. Data presented as mean ± SEM, analysed by unpaired t‐test, *p < 0.05. ns = not significant.
**Figure S7:** Phenotypes of Bmal1MKO mice and control littermates, including GTT, food intake, metabolic cage assessments, and treadmill performance. (a) GTTs for 12‐week‐old Bmal1MKO and control littermates. (b) Running distance to exhaustion on a treadmill for Bmal1MKO and control littermates. (c) Diurnal Food Intake patterns of Bmal1MKO and control littermates. (d‐f) O2 consumption, CO2 production, RER and their quantitative analysis of Bmal1MKO and control littermates. (g‐i) O2 consumption, CO2 production and RER in Bmal1MKO and control littermates during treadmill exercise. The shaded areas indicate mice under dark conditions (ZT12‐ZT24). Data presented as Mean ± SEM, analysed by unpaired t‐test, *p < 0.05.
**Figure S8:** BMAL1 does not interact with the E‐box on the Mstn promoter. (a) Schematic representation of the mouse Mstn gene promoter region, with E‐box elements highlighted in red. (b) ChIP‐qPCR analysis showing the occupancy of BMAL1 antibody‐enriched sequences near the E‐box elements of the Mstn promoter relative to input. Data presented as Mean ± SEM, analysed by unpaired t‐test, ***p < 0.001.
**Figure S9:** DBP does not interact with the D‐box on the Mstn promoter. (a) Schematic of the Mstn gene promoter region in mice, with D‐box elements highlighted in yellow. (b) Validation of DBP overexpression in C2C12 cells. (c) ChIP‐qPCR analysis showing the occupancy of FLAG antibody‐enriched sequences near the D‐box elements of the Mstn promoter relative to input. (d) The protein expression levels of MSTN in C2C12 cell lines overexpressing DBP and in negative controls. Data presented as Mean ± SEM, analysed by unpaired t‐test, ***p < 0.001.
**Figure S10:** Analysis of histone modifying enzyme expression and H3K27ac levels in skeletal muscle with disrupted circadian clock. (a) RNA‐seq analysis of TA tissue from Bmal1MKO and control littermates. (b) mRNA expression patterns of Kat14 and Kat5 in TA tissue from Bmal1MKO and control littermates at different time points (*n* = 3–4/time point). (c) Levels of H3K27ac modification in TA tissue of Bmal1MKO, Per1/Per2 DKO and their control littermates at different time points. Data presented as Mean ± SEM.

**Table S1:** Collection information of human skeletal muscle samples.

**Table S2:** RNA‐seq analysis of skeletal muscle from Bmal1MKO and WT mice.

**Table S3:** ChIP‐seq analysis of BMAL1 in mouse skeletal muscle tissue.

**Table S4:** Circadian analysis of Mstn expression across species by JTK_CYCLE.

**Data S1:** Supporting information.

## References

[1] C. Dibner , U. Schibler , and U. Albrecht , “The Mammalian Circadian Timing System: Organization and Coordination of Central and Peripheral Clocks,” Annual Review of Physiology 72, no. 1 (2010): 517–549. 10.1146/annurev-physiol-021909-135821 20148687

[2] J. Bass , “Circadian Topology of Metabolism,” Nature 491, no. 7424 (2012): 348–356. 23151577 10.1038/nature11704

[3] S. Zhang , M. Dai , X. Wang , et al., “Signalling Entrains the Peripheral Circadian Clock,” Cellular Signalling 69 (2020): 109433. 31982551 10.1016/j.cellsig.2019.109433

[4] J. S. Takahashi , “Transcriptional Architecture of the Mammalian Circadian Clock,” Nature Reviews Genetics 18, no. 3 (2017): 164–179. 10.1038/nrg.2016.150 PMC5501165 27990019

[5] R. A. Martin , M. R. Viggars , and K. A. Esser , “Metabolism and Exercise: The Skeletal Muscle Clock Takes Centre Stage,” Nature Reviews Endocrinology 19, no. 5 (2023): 272–284. 10.1038/s41574-023-00805-8 PMC11783692 36726017

[6] B. H. Miller , E. L. McDearmon , S. Panda , et al., “Circadian and CLOCK‐Controlled Regulation of the Mouse Transcriptome and Cell Proliferation,” Proceedings of the National Academy of Sciences of the United States of America 104, no. 9 (2007): 3342–3347. 17360649 10.1073/pnas.0611724104 PMC1802006

[7] J. J. McCarthy , J. L. Andrews , E. L. McDearmon , et al., “Identification of the Circadian Transcriptome in Adult Mouse Skeletal Muscle,” Physiological Genomics 31, no. 1 (2007): 86–95. 17550994 10.1152/physiolgenomics.00066.2007 PMC6080860

[8] R. Huang , J. Chen , M. Zhou , et al., “Multi‐Omics Profiling Reveals Rhythmic Liver Function Shaped by Meal Timing,” Nature Communications 14, no. 1 (2023): 6086. 10.1038/s41467-023-41759-9 PMC10541894 37773240

[9] A. Pizarro , K. Hayer , N. F. Lahens , and J. B. Hogenesch , “CircaDB: A Database of Mammalian Circadian Gene Expression Profiles,” Nucleic Acids Research 41, no. D1 (2012): D1009–D1013. 23180795 10.1093/nar/gks1161 PMC3531170

[10] H. Hoppeler and M. Flück , “Normal Mammalian Skeletal Muscle and Its Phenotypic Plasticity,” Journal of Experimental Biology 205, no. 15 (2002): 2143–2152. 12110647 10.1242/jeb.205.15.2143

[11] W. R. Frontera and J. Ochala , “Skeletal Muscle: A Brief Review of Structure and Function,” Calcified Tissue International 96 (2015): 183–195. 25294644 10.1007/s00223-014-9915-y

[12] S. V. Brooks , S. D. Guzman , and L. P. Ruiz , “Skeletal Muscle Structure, Physiology, and Function,” Handbook of Clinical Neurology 195 (2023): 3–16. 37562874 10.1016/B978-0-323-98818-6.00013-3

[13] C. A. Henderson , C. G. Gomez , S. M. Novak , L. Mi‐Mi , and C. C. Gregorio , “Overview of the Muscle Cytoskeleton,” Comprehensive Physiology 7, no. 3 (2017): 891–944. 28640448 10.1002/cphy.c160033 PMC5890934

[14] G.‐W. Li , D. Burkhardt , C. Gross , and J. S. Weissman , “Quantifying Absolute Protein Synthesis Rates Reveals Principles Underlying Allocation of Cellular Resources,” Cell 157, no. 3 (2014): 624–635. 24766808 10.1016/j.cell.2014.02.033 PMC4006352

[15] F. Atger , C. Gobet , J. Marquis , et al., “Circadian and Feeding Rhythms Differentially Affect Rhythmic mRNA Transcription and Translation in Mouse Liver,” Proceedings of the National Academy of Sciences of the United States of America 112, no. 47 (2015): E6579–E6588. 26554015 10.1073/pnas.1515308112 PMC4664316

[16] P. Janich , A. B. Arpat , V. Castelo‐Szekely , M. Lopes , and D. Gatfield , “Ribosome Profiling Reveals the Rhythmic Liver Translatome and Circadian Clock Regulation by Upstream Open Reading Frames,” Genome Research 25, no. 12 (2015): 1848–1859. 26486724 10.1101/gr.195404.115 PMC4665006

[17] J. O. Lipton , E. D. Yuan , L. M. Boyle , et al., “The Circadian Protein BMAL1 Regulates Translation in Response to S6K1‐Mediated Phosphorylation,” Cell 161, no. 5 (2015): 1138–1151. 25981667 10.1016/j.cell.2015.04.002 PMC4447213

[18] S. Schiaffino , K. A. Dyar , S. Ciciliot , B. Blaauw , and M. Sandri , “Mechanisms Regulating Skeletal Muscle Growth and Atrophy,” FEBS Journal 280, no. 17 (2013): 4294–4314. 23517348 10.1111/febs.12253

[19] P. A. Bilodeau , E. S. Coyne , and S. S. Wing , “The Ubiquitin Proteasome System in Atrophying Skeletal Muscle: Roles and Regulation,” American Journal of Physiology‐Cell Physiology 311, no. 3 (2016): C392–C403. 27510905 10.1152/ajpcell.00125.2016

[20] H. Zhang , J. Liang , and N. Chen , “Do Not Neglect the Role of Circadian Rhythm in Muscle Atrophy,” Ageing Research Reviews 63 (2020): 101155. 32882420 10.1016/j.arr.2020.101155

[21] A. C. McPherron , A. M. Lawler , and S.‐J. Lee , “Regulation of Skeletal Muscle Mass in Mice by a New TGF‐P Superfamily Member,” Nature 387, no. 6628 (1997): 83–90. 9139826 10.1038/387083a0

[22] A. C. McPherron and S.‐J. Lee , “Double Muscling in Cattle due to Mutations in the Myostatin Gene,” Proceedings of the National Academy of Sciences of the United States of America 94, no. 23 (1997): 12457–12461. 9356471 10.1073/pnas.94.23.12457 PMC24998

[23] J. Rodriguez , B. Vernus , I. Chelh , et al., “Myostatin and the Skeletal Muscle Atrophy and Hypertrophy Signaling Pathways,” Cellular and Molecular Life Sciences 71 (2014): 4361–4371. 25080109 10.1007/s00018-014-1689-x PMC11113773

[24] J. P. Gumucio and C. L. Mendias , “Atrogin‐1, MuRF‐1, and Sarcopenia,” Endocrine Reviews 43 (2013): 12–21. 10.1007/s12020-012-9751-7 PMC3586538 22815045

[25] A. U. Trendelenburg , A. Meyer , D. Rohner , J. Boyle , S. Hatakeyama , and D. J. Glass , “Myostatin Reduces Akt/TORC1/p70S6K Signaling, Inhibiting Myoblast Differentiation and Myotube Size,” American Journal of Physiology‐Cell Physiology 296, no. 6 (2009): C1258–C1270. 19357233 10.1152/ajpcell.00105.2009

[26] S.‐J. Lee , “Myostatin: A Skeletal Muscle Chalone,” Annual Review of Physiology 85, no. 1 (2023): 269–291. 10.1146/annurev-physiol-012422-112116 PMC10163667 36266260

[27] D. L. Helterline , D. Garikipati , D. L. Stenkamp , and B. D. Rodgers , “Embryonic and Tissue‐Specific Regulation of Myostatin‐1 and‐2 Gene Expression in Zebrafish,” General and Comparative Endocrinology 151, no. 1 (2007): 90–97. 17289047 10.1016/j.ygcen.2006.12.023 PMC2586822

[28] C. Xu , G. Wu , Y. Zohar , and S.‐J. Du , “Analysis of Myostatin Gene Structure, Expression and Function in Zebrafish,” Journal of Experimental Biology 206, no. 22 (2003): 4067–4079. 14555747 10.1242/jeb.00635

[29] C. Wang , Y.‐L. Chen , W.‐P. Bian , et al., “Deletion of Mstna and Mstnb Impairs the Immune System and Affects Growth Performance in Zebrafish,” Fish Shellfish Immunology 72 (2018): 572–580. 29175471 10.1016/j.fsi.2017.11.040

[30] B. D. Harfmann , E. A. Schroder , M. T. Kachman , B. A. Hodge , X. Zhang , and K. A. J. S. Esser , “Muscle‐Specific Loss of Bmal1 Leads to Disrupted Tissue Glucose Metabolism and Systemic Glucose Homeostasis,” Skeletal Muscle 6, no. 1 (2016): 12. 27486508 10.1186/s13395-016-0082-x PMC4969979

[31] K. Sivakumar , T. I. Cochrane , B. Sloth , et al., “Long‐Term Safety and Tolerability of Bimagrumab (BYM338) in Sporadic Inclusion Body Myositis,” Neurology 95, no. 14 (2020): e1971–e1978. 32690797 10.1212/WNL.0000000000010417

[32] M. I. Polkey , J. Praestgaard , A. Berwick , et al., “Activin Type II Receptor Blockade for Treatment of Muscle Depletion in Chronic Obstructive Pulmonary Disease. A Randomized Trial,” American Journal of Respiratory and Critical Care Medicine 199, no. 3 (2019): 313–320. 30095981 10.1164/rccm.201802-0286OC PMC6363975

[33] R. Wang , F. Chen , Q. Chen , et al., “MyoD Is a 3D Genome Structure Organizer for Muscle Cell Identity,” Nature Communications 13, no. 1 (2022): 205. 10.1038/s41467-021-27865-6 PMC8752600 35017543

[34] C. V. C. Grade , C. S. Mantovani , and L. E. Alvares , “Myostatin Gene Promoter: Structure, Conservation and Importance as a Target for Muscle Modulation,” Journal of Animal Science and Biotechnology 10 (2019): 1–19. 31044074 10.1186/s40104-019-0338-5 PMC6477727

[35] X. Kong , T. Yao , P. Zhou , et al., “Brown Adipose Tissue Controls Skeletal Muscle Function via the Secretion of Myostatin,” Cell Metabolism 28, no. 4 (2018): 631–643.e633 30078553 10.1016/j.cmet.2018.07.004 PMC6170693

[36] H. Wang , S. Guo , H. Gao , et al., “Myostatin Regulates Energy Homeostasis Through Autocrine‐and Paracrine‐Mediated Microenvironment Communications,” Journal of Clinical Investigation 134 (2024): e178303. 38889010 10.1172/JCI178303 PMC11324308

[37] T. Shan , X. Liang , P. Bi , and S. Kuang , “Myostatin Knockout Drives Browning of White Adipose Tissue Through Activating the AMPK‐PGC1α‐Fndc5 Pathway in Muscle,” FASEB Journal 27, no. 5 (2013): 1981–1989. 23362117 10.1096/fj.12-225755 PMC3633817

[38] A. C. McPherron and S.‐J. J. T. J. Lee , “Suppression of Body Fat Accumulation in Myostatin‐Deficient Mice,” Journal of Clinical Investigation 109, no. 5 (2002): 595–601. 11877467 10.1172/JCI13562 PMC150888

[39] H. Xin , R. Huang , M. Zhou , et al., “Daytime‐Restricted Feeding Enhances Running Endurance Without Prior Exercise in Mice,” Nature Metabolism 5, no. 7 (2023): 1236–1251. 10.1038/s42255-023-00826-7 37365376

[40] C. M. Douglas , S. J. Hesketh , and K. A. Esser , “Time of Day and Muscle Strength: A Circadian Output?,” Physiology 36, no. 1 (2021): 44–51. 33325817 10.1152/physiol.00030.2020 PMC8425416

