# Supplementary material for: Myostatin Exhibits an Evolutionarily Conserved Circadian Pattern in Skeletal Muscles
Source: J Cachexia Sarcopenia Muscle. 2025 Nov 24;16(6):e70130. doi: 10.1002/jcsm.70130 (PMC12644245; doi:10.1002/jcsm.70130)
Supplement: Supplementary file 1 — Figure S1: A complete circadian clock system is present in the skeletal muscle of mice. The mRNA expression patterns of key clock genes such as per1, clock, and cry1 at different time points in the skeletal muscle of B6J mice. The rhythmicity of gene expression was assessed using JTK analysis, with p < 0.05 considered rhythmic. Data presented as Mean ± SEM. Figure S2: Circadian rhythms in mice are disrupted under LL conditions. Representative wheel‐running data for B6J mice under LL conditions. Figure S3: The mRNA expression of mstn at different time points in muscle tissues of Cyprinus carpio, Carassius auratus and Procambarus clarkii. (a) The mRNA expression of mstn, arntl1 and clock at different time points in the muscle of Cyprinus carpio (n = 7–12/time point). (b) The mRNA expression of mstn, arntl1 and clock at different time points in the muscle of Carassius auratus (n = 5–12/time point). (c) The mRNA expression of mstn, arntl1 and clock at different time points in the muscle tissue of Procambarus clarkii (n = 10–18/time point). The rhythmicity of gene expression was assessed using JTK analysis, with p < 0.05 considered rhythmic. Data presented as mean ± SEM, analysed by unpaired t‐test with Bonferroni correction, bars sharing the same lowercase letter indicate no significant difference (p > 0.05). Figure S4: RNA‐seq revealed a significant downregulation of Mstn mRNA expression in the skeletal muscle of Bmal1MKO mice. (a)The protein expression patterns of BMAL1 at different time points in the skeletal muscle of B6J mice. (b) RNA‐Seq volcano plot of the TA skeletal muscle in Bmal1MKO and control littermates. (c) Heatmap showing the mRNA expression of key clock genes in the TA skeletal muscle of Bmal1MKO and control littermates. (d) KEGG pathway analysis of RNA‐seq data from the TA skeletal muscle of Bmal1MKO and control littermates. (e) FPKM values of Mstn from RNA‐Seq results in the TA skeletal muscle of Bmal1MKO and control littermates. Data presented as Mea [file JCSM-16-e70130-s001.docx]

**Supplemental Data for**

**Myostatin exhibits an evolutionarily conserved circadian pattern in skeletal muscles**

Xiangpeng Liu^1, 3, #^, Changyou Song^2, #^, Yan Xiong^4, #^, Jinxin Gu^5, #^, Lianxin Wu^6^, Taole Liu^6^, Xiyue Chen^7^, Hui Shu^1^, Yingying Dong^1^, Tizhong Shan^8^, Sheng Wang^9^, Yucheng Zhu^10^, Tongxing Song^10^, Lei Fu^5^, Yaqiu Lin^4^, Can Liu^11^, Ruiqi Zheng^12^, Xiao Zhao^12^, Hongxia Li^3^, Yong Xu^13^, Shihuan Kuang^7, 14^, Han Wang^6,^ ^****^, Bin Guo^5,^ ^***^, Pao Xu^2,^ ^**^, Zhihao Jia^1, *, $^

^1^Cambridge-Suda Genomic Resource Center, Suzhou Medical College, Soochow University, Suzhou 215000, China.

^2^Freshwater Fisheries Research Center, Chinese Academy of Fishery Sciences, Wuxi, 214081, China

^3^Institues of Biomedical Sciences, Inner Mongolia University, Hohhot 010000, China

^4^Key Laboratory of Qinghai-Tibetan Plateau Animal Genetic Resource Reservation and Utilization, Ministry of Education, Southwest Minzu University, Chengdu, 610041, China

^5^Wisdom Lake Academy of Pharmacy, Xi'an Jiaotong-Liverpool University, Suzhou 215000, China.

^6^Center for Circadian Clocks, Soochow University, and School of Basic Medical Sciences, Suzhou Medical College, Suzhou 215000, Jiangsu, China

^7^Department of Animal Sciences, Purdue University, West Lafayette, Indiana, 47907, USA.

^8^College of Animal Sciences, Zhejiang University, Hangzhou, 310058, China

^9^State Key Laboratory of Biocontrol, School of Life Sciences, Sun Yat-sen University, Guangzhou, China

^10^College of Animal Science and Technology, Huazhong Agricultural University, Wuhan 430070, China

^11^Departmen of Orthopedics, The Third Affiliated Hospital of Southern Medical University, Guangzhou 510630, China

^12^Med-X institute, Center for Immunological and Metabolic Diseases and Department of Endocrinology, First Affiliated Hospital of Xi'an Jiaotong University, Xi'an Jiaotong University, Xi’an, China

^13^Orthopaedic Institute, Suzhou Medical College, Soochow University, Suzhou 215000, China

^14^Department of Orthopedic Surgery and Department of Cell Biology, Duke University School of Medicine, Durham, NC 27710, USA.





**Fig. S1. A complete circadian clock system is present in the skeletal muscle of mice.** The mRNA expression patterns of key clock genes such as *per1*, *clock*, and *cry1* at different time points in the skeletal muscle of B6J mice. The rhythmicity of gene expression was assessed using JTK analysis, with *p* < 0.05 considered rhythmic. Data presented as Mean ± SEM.


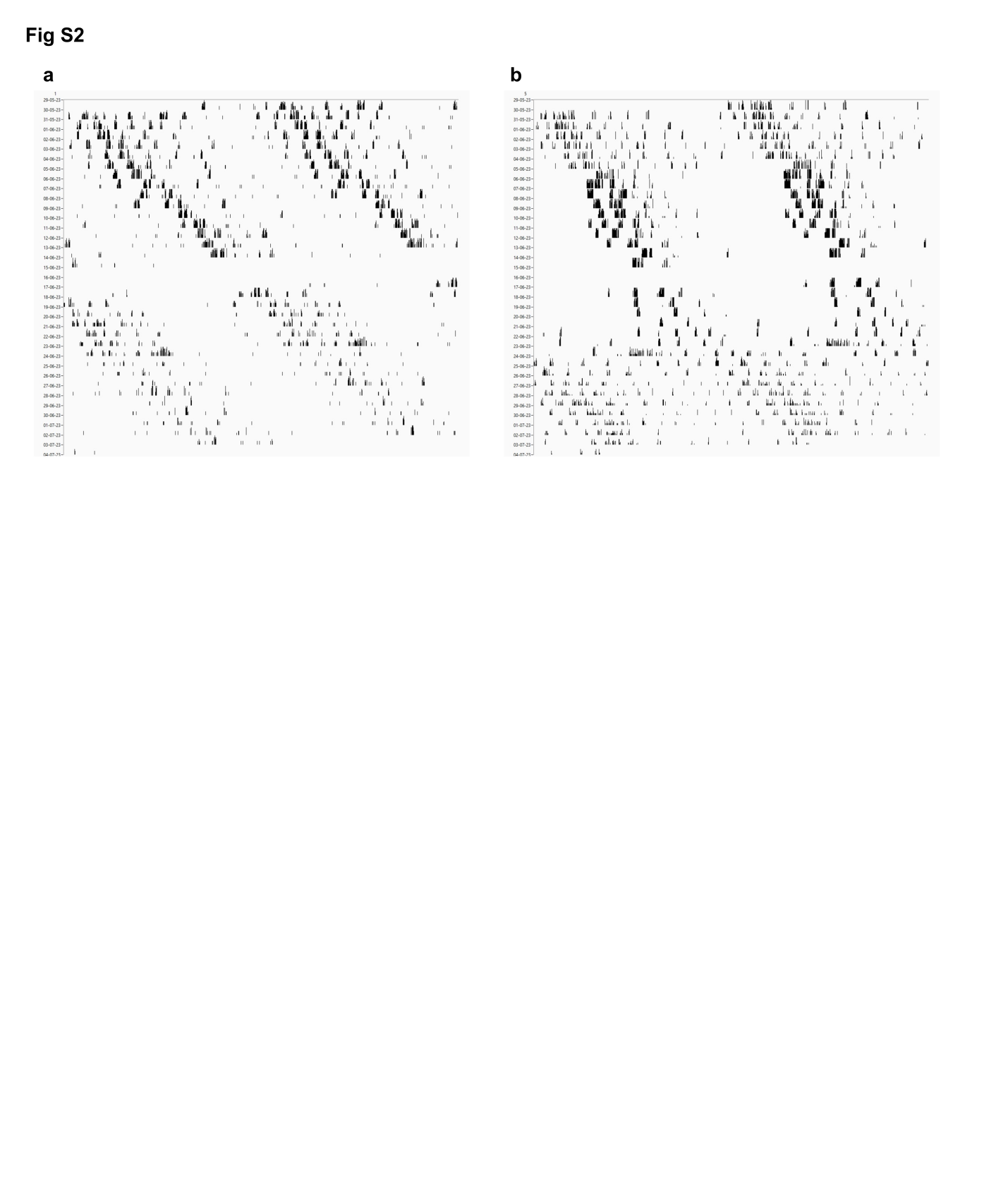


Fig. S2. Circadian rhythms in mice are disrupted under LL conditions. Representative wheel-running data for B6J mice under LL conditions.


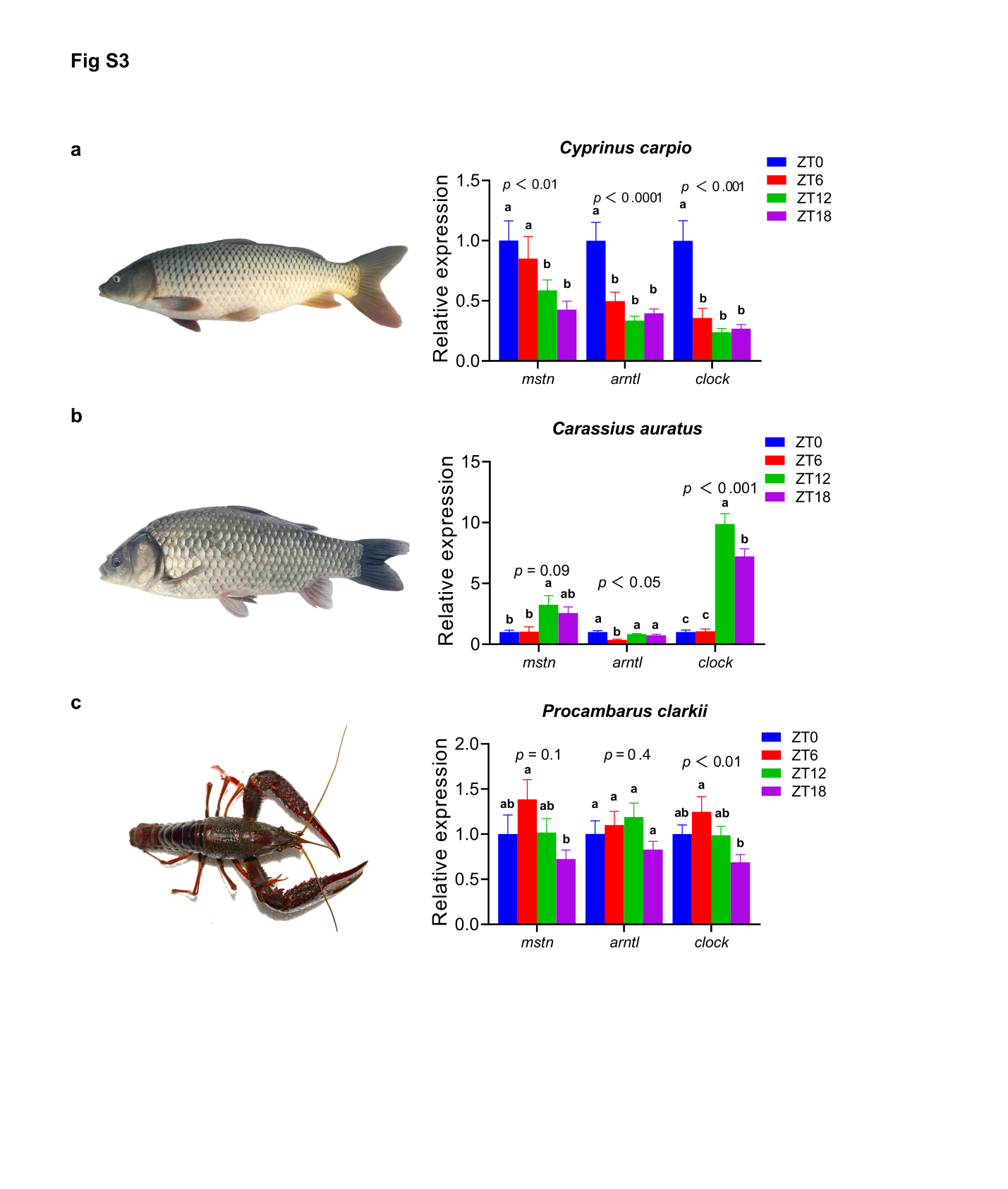


**Fig. S3.** **The mRNA expression of *mstn* at different time points in muscle tissues of *Cyprinus carpio*, *Carassius auratus* and *Procambarus clarkii*.** (a) The mRNA expression of *mstn*, *arntl1* and *clock* at different time points in the muscle of *Cyprinus carpio* (n=7-12/time point). (b) The mRNA expression of *mstn*, *arntl1* and *clock* at different time points in the muscle of *Carassius auratus* (n=5-12/time point). (c) The mRNA expression of *mstn*, *arntl1* and *clock* at different time points in the muscle tissue of *Procambarus clarkii* (n=10-18/time point). The rhythmicity of gene expression was assessed using JTK analysis, with *p* < 0.05 considered rhythmic. Data presented as mean ± SEM, analyzed by unpaired t-test with Bonferroni correction, bars sharing the same lowercase letter indicate no significant difference (*p* > 0.05).


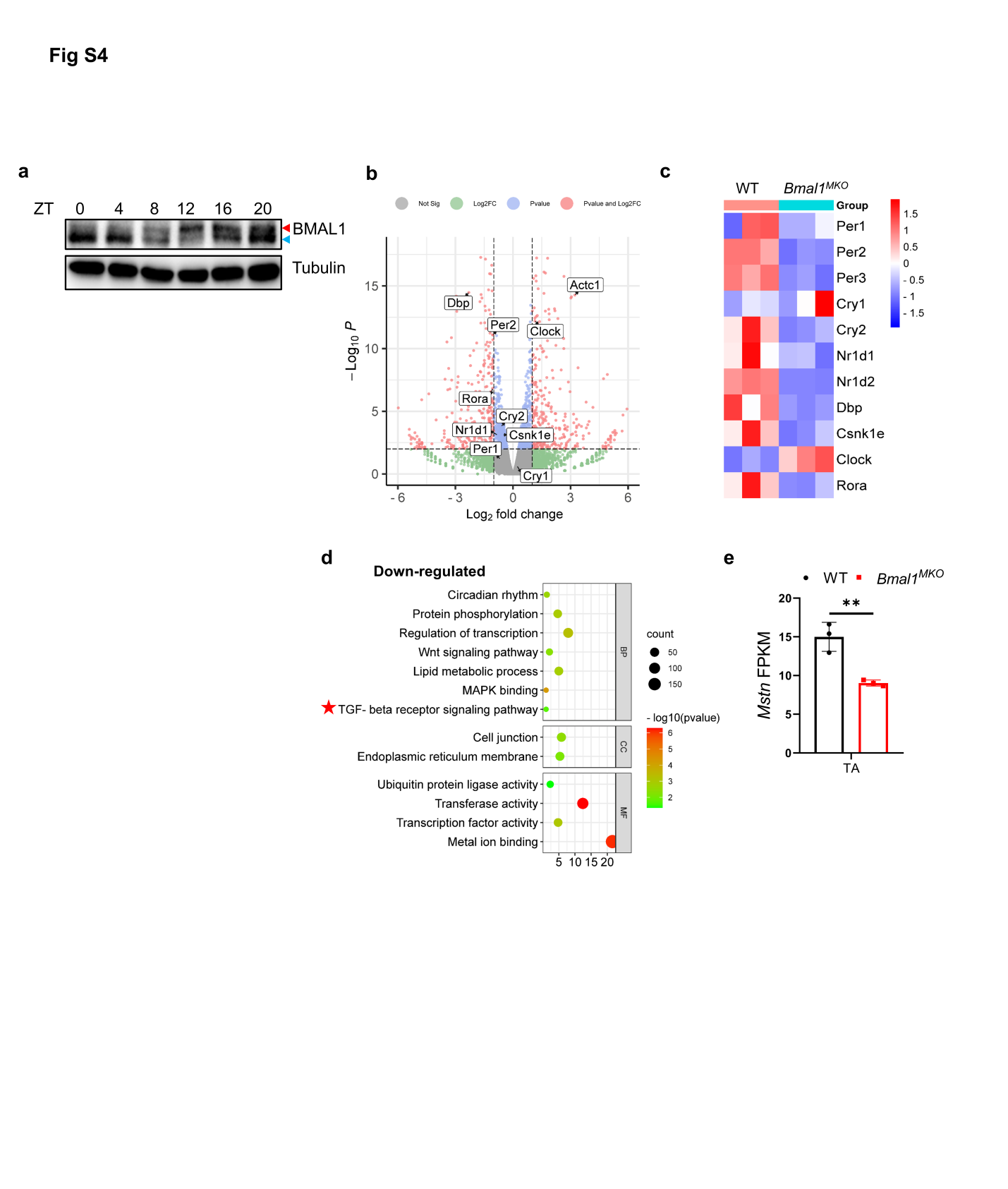


Fig. S4. RNA-seq revealed a significant downregulation of *Mstn* mRNA expression in the skeletal muscle of *Bmal1^MKO^* mice. (a)The protein expression patterns of BMAL1 at different time points in the skeletal muscle of B6J mice. (b) RNA-Seq volcano plot of the TA skeletal muscle in *Bmal1^MKO^* and control littermates. (c) Heatmap showing the mRNA expression of key clock genes in the TA skeletal muscle of *Bmal1^MKO^* and control littermates. (d) KEGG pathway analysis of RNA-seq data from the TA skeletal muscle of *Bmal1^MKO^* and control littermates. (e) FPKM values of *Mstn* from RNA-Seq results in the TA skeletal muscle of *Bmal1^MKO^* and control littermates. Data presented as Mean ± SEM, analyzed by unpaired t-test, ***p* < 0.01.

**
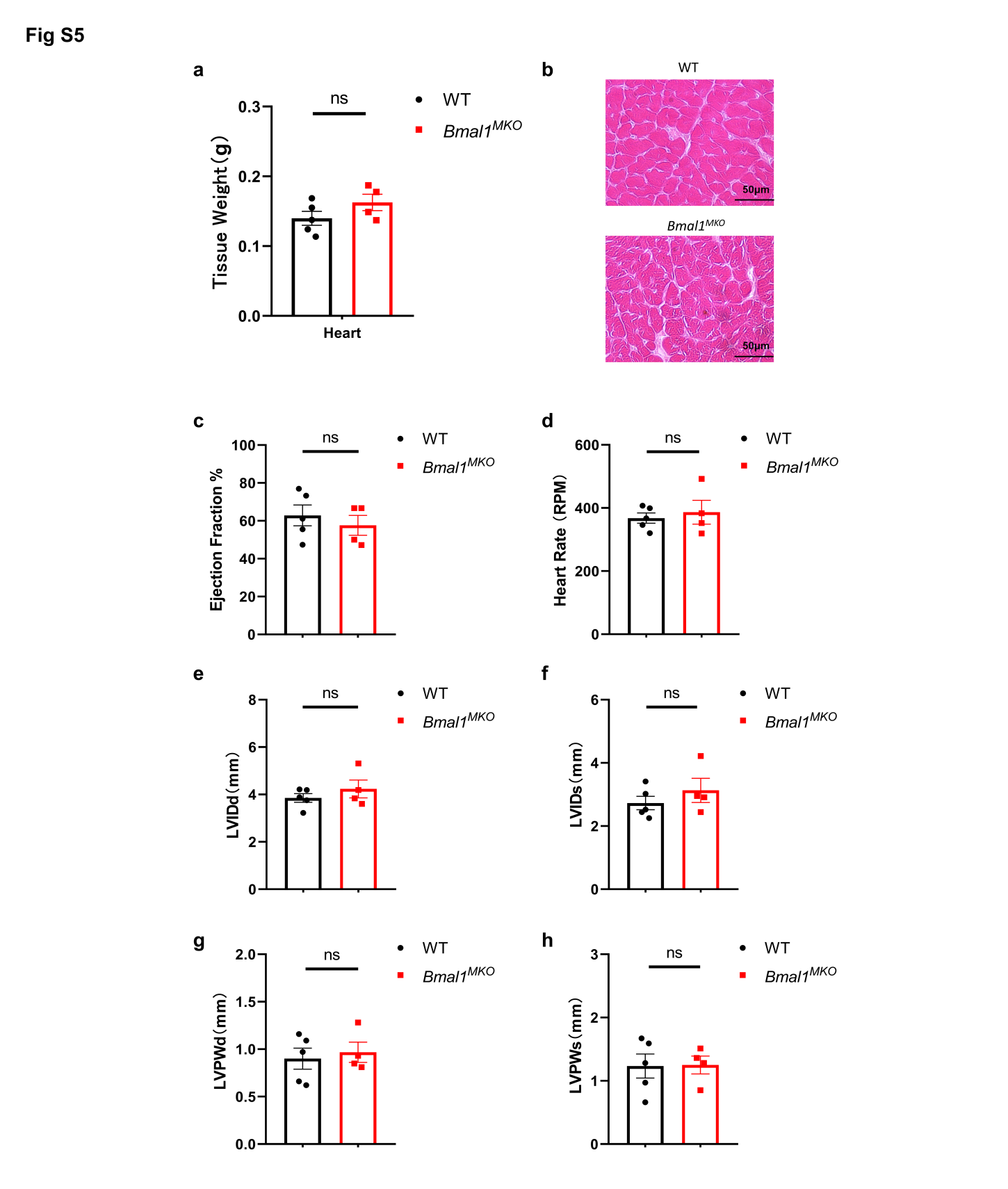
**

**Fig. S5.** **Cardiac morphology and function are comparable between *Bmal1^MKO^* and control littermates.** (a) Heart weight of 12-week-old *Bmal1^MKO^* and control littermates. (b) Representative HE-stained myocardial sections of *Bmal1^MKO^* and control littermates. Scale bar:50μm. (c-h) Echocardiographic assessment of cardiac function parameters: (c) Ejection fraction (EF), (d) Heart rate, (e) Left ventricular internal diameter in diastole (LVIDd), (f) Left ventricular internal diameter in systole (LVIDs), (g) Left ventricular posterior wall thickness in diastole (LVPWd), and (h) Left ventricular posterior wall thickness in systole (LVPWs). Data presented as Mean ± SEM (n=4-5/group), analyzed by unpaired t-test, ns = not significant.





Fig. S6. *Bmal1^MKO^* mice exhibit increased expression of AKT and pAKT in skeletal muscle. (a-b) Protein expression levels and grayscale quantification of AKT, pAKT and pAKT/AKT in TA tissue of *Bmal1^MKO^* and control littermates. (c) Protein expression levels of AKT and pAKT at different time points in TA tissue of *Bmal1^MKO^* and control littermates. Data presented as Mean ± SEM, analyzed by unpaired t-test, **p* < 0.05. ns = not significant.





Fig. S7. Phenotypes of *Bmal1^MKO^* mice and control littermates, including GTT, food intake, metabolic cage assessments, and treadmill performance. (a) GTTs for 12-week-old *Bmal1^MKO^* and control littermates. (b) Running distance to exhaustion on a treadmill for *Bmal1^MKO^* and control littermates. (c) Diurnal Food Intake patterns of *Bmal1^MKO^* and control littermates. (d-f) O_2_ consumption, CO_2_ production, RER and their quantitative analysis of *Bmal1^MKO^* and control littermates. (g-i) O_2_ consumption, CO_2_ production and RER in *Bmal1^MKO^* and control littermates during treadmill exercise. The shaded areas indicate mice under dark conditions (ZT12-ZT24). Data presented as Mean ± SEM, analyzed by unpaired t-test, **p* < 0.05.





Fig. S8. BMAL1 does not interact with the E-box on the *Mstn* promoter. (a) Schematic representation of the mouse Mstn gene promoter region, with E-box elements highlighted in red. (b) ChIP-qPCR analysis showing the occupancy of BMAL1 antibody-enriched sequences near the E-box elements of the *Mstn* promoter relative to input. Data presented as Mean ± SEM, analyzed by unpaired t-test, ****p* < 0.001.


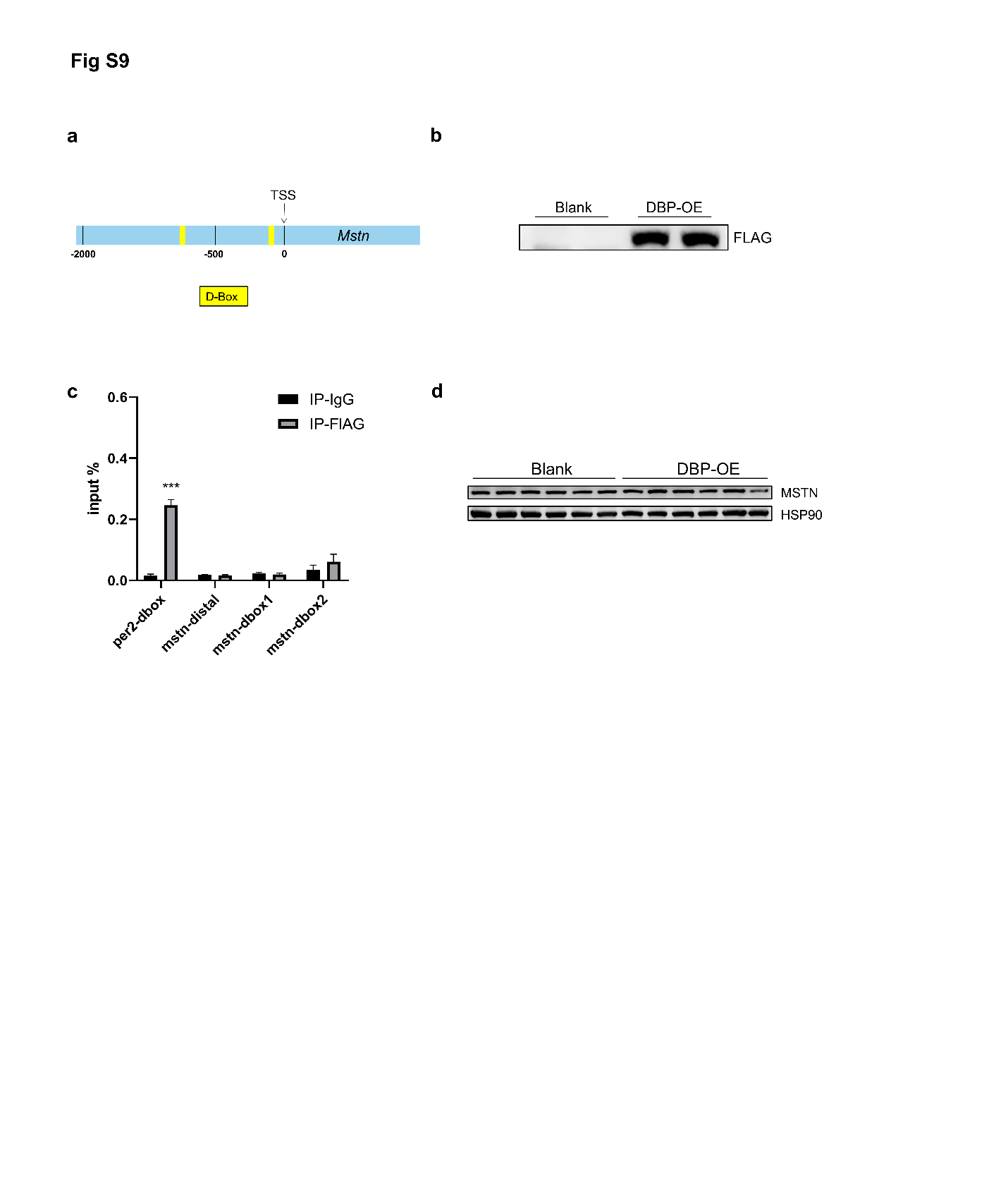


Fig. S9. DBP does not interact with the D-box on the *Mstn* promoter. (a) Schematic of the Mstn gene promoter region in mice, with D-box elements highlighted in yellow. (b) Validation of DBP overexpression in C2C12 cells. (c) ChIP-qPCR analysis showing the occupancy of FLAG antibody-enriched sequences near the D-box elements of the *Mstn* promoter relative to input. (d) The protein expression levels of MSTN in C2C12 cell lines overexpressing DBP and in negative controls. Data presented as Mean ± SEM, analyzed by unpaired t-test, ****p* < 0.001.


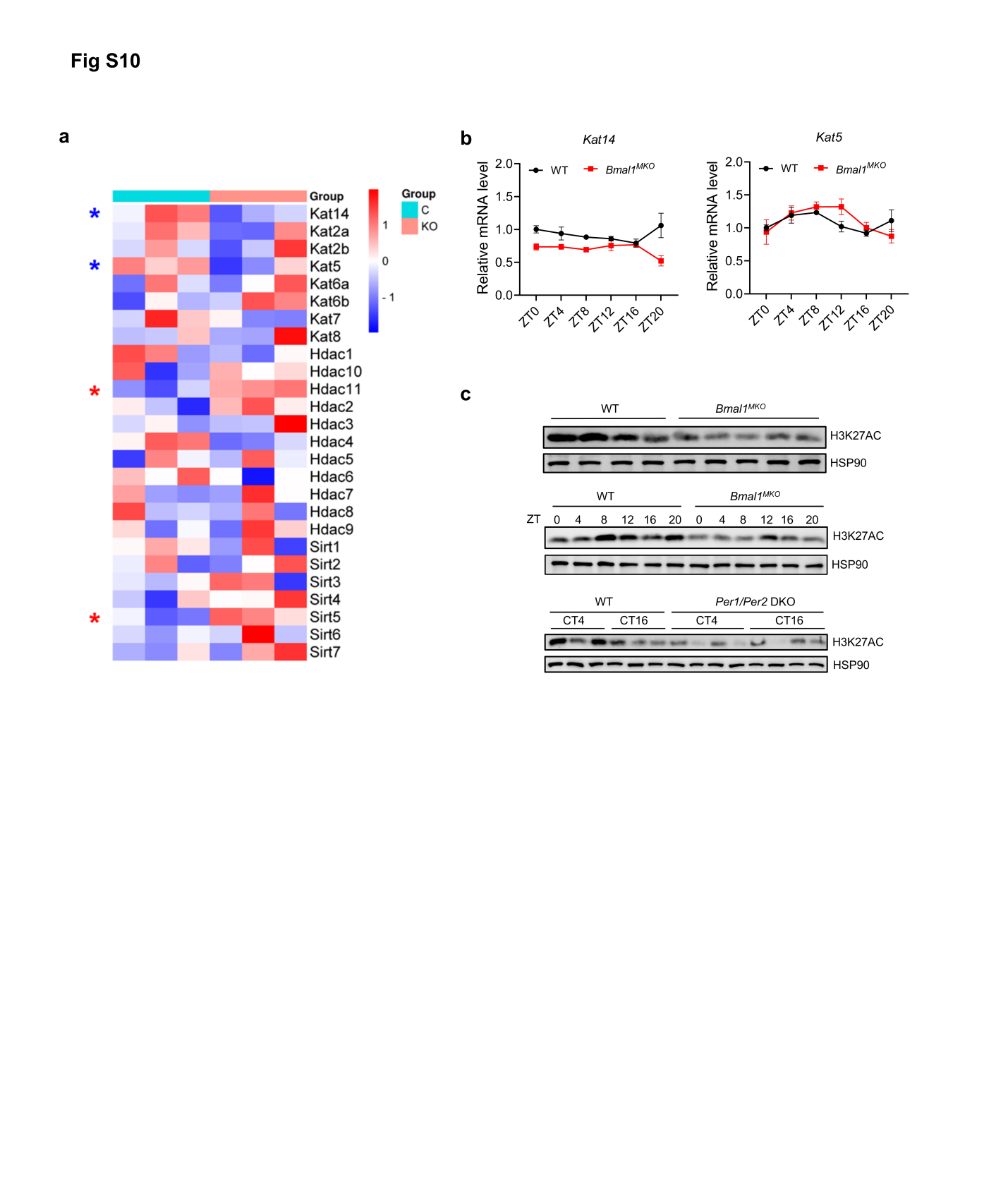


**Fig. S10. Analysis of histone modifying enzyme expression and H3K27ac levels in skeletal muscle with disrupted circadian clock.** (a) RNA-seq analysis of TA tissue from *Bmal1^MKO^* and control littermates. (b） mRNA expression patterns of *Kat14* and *Kat5* in TA tissue from *Bmal1^MKO^* and control littermates at different time points (n=3-4/time point). (c) Levels of H3K27ac modification in TA tissue of *Bmal1^MKO^*, *Per1/Per2* DKO and their control littermates at different time points. Data presented as Mean ± SEM.

**Table S1. Collection Information of Human Skeletal Muscle Samples.**

**Table S2. RNA-Seq Analysis of Skeletal Muscle from Bmal1MKO and WT Mice.**

**Table S3. ChIP-Seq Analysis of BMAL1 in Mouse Skeletal Muscle Tissue.**

**Table S4. Circadian Analysis of Mstn Expression Across Species by JTK_CYCLE.​**

**Additional reference**

[s1] Roy PJ, Stuart JM, Lund J, Kim SK (2002) Chromosomal clustering of muscle-expressed genes in Caenorhabditis elegans. Nature 418(6901): 975-979

[s2] Schiaffino S, Blaauw B, Dyar KAJSm (2016) The functional significance of the skeletal muscle clock: lessons from Bmal1 knockout models. 6(1): 33

[s3] Dyar KA, Ciciliot S, Wright LE, et al. (2014) Muscle insulin sensitivity and glucose metabolism are controlled by the intrinsic muscle clock. *Molecular metabolism*, *3*(1), 29-41.

[s4] Dyar KA, Hubert MJ, Mir AA, et al. (2018) Transcriptional programming of lipid and amino acid metabolism by the skeletal muscle circadian clock. *PLoS biology*, *16*(8), e2005886.

[s5] Suh J, Kim N-K, Lee S-H, et al. (2020) GDF11 promotes osteogenesis as opposed to MSTN, and follistatin, a MSTN/GDF11 inhibitor, increases muscle mass but weakens bone. Proceedings of the National Academy of Sciences 117(9): 4910-4920

[s6] Andrews JL, Zhang X, McCarthy JJ, et al. (2010) CLOCK and BMAL1 regulate MyoD and are necessary for maintenance of skeletal muscle phenotype and function. Proceedings of the National Academy of Sciences 107(44): 19090-19095

[s7] Spiller MP, Kambadur R, Jeanplong F, et al. (2002) The myostatin gene is a downstream target gene of basic helix-loop-helix transcription factor MyoD. Molecular cellular biology

[s8] Ueda HR, Hayashi S, Chen W, et al. (2005) System-level identification of transcriptional circuits underlying mammalian circadian clocks. Nature genetics 37(2): 187-192

[s9] Yoshitane H, Asano Y, Sagami A, et al. (2019) Functional D-box sequences reset the circadian clock and drive mRNA rhythms. Communications Biology 2(1): 300

[s10] An Y, Yuan B, Xie P, et al. (2022) Decoupling PER phosphorylation, stability and rhythmic expression from circadian clock function by abolishing PER-CK1 interaction. 13(1): 3991

[s11] Ben-Moshe Livne Z, Alon S, Vallone D, et al. (2016) Genetically blocking the zebrafish pineal clock affects circadian behavior. PLoS genetics 12(11): e1006445

[s12] Harfmann BD, Schroder EA, Esser KA (2015) Circadian rhythms, the molecular clock, and skeletal muscle. Journal of biological rhythms 30(2): 84-94

[s13] Lee S-J (2021) Targeting the myostatin signaling pathway to treat muscle loss and metabolic dysfunction. The Journal of clinical investigation 131(9)
