# Supplementary material for: Myostatin Exhibits an Evolutionarily Conserved Circadian Pattern in Skeletal Muscles
Source: J Cachexia Sarcopenia Muscle. 2025 Nov 24;16(6):e70130. doi: 10.1002/jcsm.70130 (PMC12644245; doi:10.1002/jcsm.70130)
Supplement: Supplementary file 6 — Data S1: Supporting information. [file JCSM-16-e70130-s006.docx]

**Fig. S1. A complete circadian clock system is present in the skeletal muscle of mice.** The mRNA expression patterns of key clock genes such as *per1*, *clock*, and *cry1* at different time points in the skeletal muscle of B6J mice. The rhythmicity of gene expression was assessed using JTK analysis, with *p* < 0.05 considered rhythmic. Data presented as Mean ± SEM.

**Fig. S2. Circadian rhythms in mice are disrupted under LL conditions.** Representative wheel-running data for B6J mice under LL conditions.

**Fig. S3.** **The mRNA expression of *mstn* at different time points in muscle tissues of *Cyprinus carpio*, *Carassius auratus* and *Procambarus clarkii*.** (a) The mRNA expression of *mstn*, *arntl1* and *clock* at different time points in the muscle of *Cyprinus carpio* (n=7-12/time point). (b) The mRNA expression of *mstn*, *arntl1* and *clock* at different time points in the muscle of *Carassius auratus* (n=5-12/time point). (c) The mRNA expression of *mstn*, *arntl1* and *clock* at different time points in the muscle tissue of *Procambarus clarkii* (n=10-18/time point). The rhythmicity of gene expression was assessed using JTK analysis, with *p* < 0.05 considered rhythmic. Data presented as mean ± SEM, analyzed by unpaired t-test with Bonferroni correction, bars sharing the same lowercase letter indicate no significant difference (*p* > 0.05).

**Fig. S4. RNA-seq revealed a significant downregulation of *Mstn* mRNA expression in the skeletal muscle of *Bmal1^MKO^* mice.** (a)The protein expression patterns of BMAL1 at different time points in the skeletal muscle of B6J mice. (b) RNA-Seq volcano plot of the TA skeletal muscle in *Bmal1^MKO^* and control littermates. (c) Heatmap showing the mRNA expression of key clock genes in the TA skeletal muscle of *Bmal1^MKO^* and control littermates. (d) KEGG pathway analysis of RNA-seq data from the TA skeletal muscle of *Bmal1^MKO^* and control littermates. (e) FPKM values of *Mstn* from RNA-Seq results in the TA skeletal muscle of *Bmal1^MKO^* and control littermates. Data presented as Mean ± SEM, analyzed by unpaired t-test, ***p* < 0.01.

**Fig. S5.** **Cardiac morphology and function are comparable between *Bmal1^MKO^* and control littermates.** (a) Heart weight of 12-week-old *Bmal1^MKO^* and control littermates. (b) Representative HE-stained myocardial sections of *Bmal1^MKO^* and control littermates. Scale bar:50μm. (c-h) Echocardiographic assessment of cardiac function parameters: (c) Ejection fraction (EF), (d) Heart rate, (e) Left ventricular internal diameter in diastole (LVIDd), (f) Left ventricular internal diameter in systole (LVIDs), (g) Left ventricular posterior wall thickness in diastole (LVPWd), and (h) Left ventricular posterior wall thickness in systole (LVPWs). Data presented as Mean ± SEM (n=4-5/group), analyzed by unpaired t-test, ns = not significant.

Fig. S6. *Bmal1^MKO^* mice exhibit increased expression of AKT and pAKT in skeletal muscle. (a-b) Protein expression levels and grayscale quantification of AKT, pAKT and pAKT/AKT in TA tissue of *Bmal1^MKO^* and control littermates. (c) Protein expression levels of AKT and pAKT at different time points in TA tissue of *Bmal1^MKO^* and control littermates. Data presented as Mean ± SEM, analyzed by unpaired t-test, **p* < 0.05. ns = not significant.

Fig. S7. Phenotypes of *Bmal1^MKO^* mice and control littermates, including GTT, food intake, metabolic cage assessments, and treadmill performance. (a) GTTs for 12-week-old *Bmal1^MKO^* and control littermates. (b) Running distance to exhaustion on a treadmill for *Bmal1^MKO^* and control littermates. (c) Diurnal Food Intake patterns of *Bmal1^MKO^* and control littermates. (d-f) O_2_ consumption, CO_2_ production, RER and their quantitative analysis of *Bmal1^MKO^* and control littermates. (g-i) O_2_ consumption, CO_2_ production and RER in *Bmal1^MKO^* and control littermates during treadmill exercise. The shaded areas indicate mice under dark conditions (ZT12-ZT24). Data presented as Mean ± SEM, analyzed by unpaired t-test, **p* < 0.05.

**Fig. S8. BMAL1 does not interact with the E-box on the *Mstn* promoter.** (a) Schematic representation of the mouse Mstn gene promoter region, with E-box elements highlighted in red. (b) ChIP-qPCR analysis showing the occupancy of BMAL1 antibody-enriched sequences near the E-box elements of the *Mstn* promoter relative to input. Data presented as Mean ± SEM, analyzed by unpaired t-test, ****p* < 0.001.

Fig. S9. DBP does not interact with the D-box on the *Mstn* promoter. (a) Schematic of the Mstn gene promoter region in mice, with D-box elements highlighted in yellow. (b) Validation of DBP overexpression in C2C12 cells. (c) ChIP-qPCR analysis showing the occupancy of FLAG antibody-enriched sequences near the D-box elements of the *Mstn* promoter relative to input. (d) The protein expression levels of MSTN in C2C12 cell lines overexpressing DBP and in negative controls. Data presented as Mean ± SEM, analyzed by unpaired t-test, ****p* < 0.001.

**Fig. S10. Analysis of histone modifying enzyme expression and H3K27ac levels in skeletal muscle with disrupted circadian clock.** (a) RNA-seq analysis of TA tissue from *Bmal1^MKO^* and control littermates. (b） mRNA expression patterns of *Kat14* and *Kat5* in TA tissue from *Bmal1^MKO^* and control littermates at different time points (n=3-4/time point). (c) Levels of H3K27ac modification in TA tissue of *Bmal1^MKO^*, *Per1/Per2* DKO and their control littermates at different time points. Data presented as Mean ± SEM.

**Table S1. Collection Information of Human Skeletal Muscle Samples.**

**Table S2. RNA-Seq Analysis of Skeletal Muscle from Bmal1MKO and WT Mice.**

**Table S3. ChIP-Seq Analysis of BMAL1 in Mouse Skeletal Muscle Tissue.**

**Table S4. Circadian Analysis of Mstn Expression Across Species by JTK_CYCLE.​**
